# Visible Light Photoredox Catalysis in the Synthesis of Phosphonate-Substituted 1,10-Phenanthrolines

**DOI:** 10.3390/molecules29235558

**Published:** 2024-11-25

**Authors:** Gleb V. Morozkov, Artem A. Troickiy, Alexei D. Averin, Alexander Yu. Mitrofanov, Anton S. Abel, Irina P. Beletskaya

**Affiliations:** 1 Department of Chemistry, Lomonosov Moscow State University, Leninskie Gory 1-3, Moscow 119991, Russia; gmorozkov@gmail.com (G.V.M.); troitskiy.a.a@yandex.ru (A.A.T.); alexaveron@yandex.ru (A.D.A.); beletska@org.chem.msu.ru (I.P.B.); 2 Frumkin Institute of Physical Chemistry and Electrochemistry, Russian Academy of Sciences, Leninsky Pr. 31, Moscow 119071, Russia

**Keywords:** phosphonylation, 1,10-phenanthroline, photocatalysis, organic photocatalysts, triethyl phosphite

## Abstract

Photoredox-catalyzed phosphonylation of bromo-substituted 1,10-phenanthrolines under visible light irradiation was studied. The reaction was shown to proceed under mild conditions with Eosin Y as a photocatalyst in DMSO under blue light irradiation. It is transition-metal-free and affords the target phosphonate-substituted 1,10-phenanthrolines in moderate yields (26–51%) in 22 to 40 h. The rate and selectivity of the reaction depend largely on the position of the bromine atom, as well as on the nature and position of other substituents in the 1,10-phenanthroline core.

## 1. Introduction

1,10-Phenanthroline and N-ligands derived from it are widely used as catalysts, photoactive systems, building blocks of supramolecular assemblies, chemosensors, etc. [1,2,3] The properties of ligands based on 1,10-phenanthroline and their complexes largely depend on the nature of the substituents and their position in the phenanthroline core, and the introduction of functional groups into various positions of 1,10-phenanthroline is a convenient approach for fine tuning of the properties of the target compound [4,5,6,7]. One of the widely used methods of 1,10-phenanthroline functionalization is cross-coupling reactions catalyzed by transition metal complexes, which, on the one hand, include the use of synthetically available halogen derivatives of 1,10-phenanthrolines, and on the other hand, provide a wide variety of introducing substituents [8,9]. A significant limitation of this approach is that 1,10-phenanthroline and its derivatives themselves are effective chelators of transition metals, which leads to the deterioration of the catalyzing species and suppression of the target reaction [8,10,11]. To overcome this difficulty, additional measures have to be taken, such as the use of expensive ligands, excess reagents, etc., which complicates the synthesis. An additional problem is the purification of the obtained products from traces of transition metals and their ligands.

**Scheme 1:**
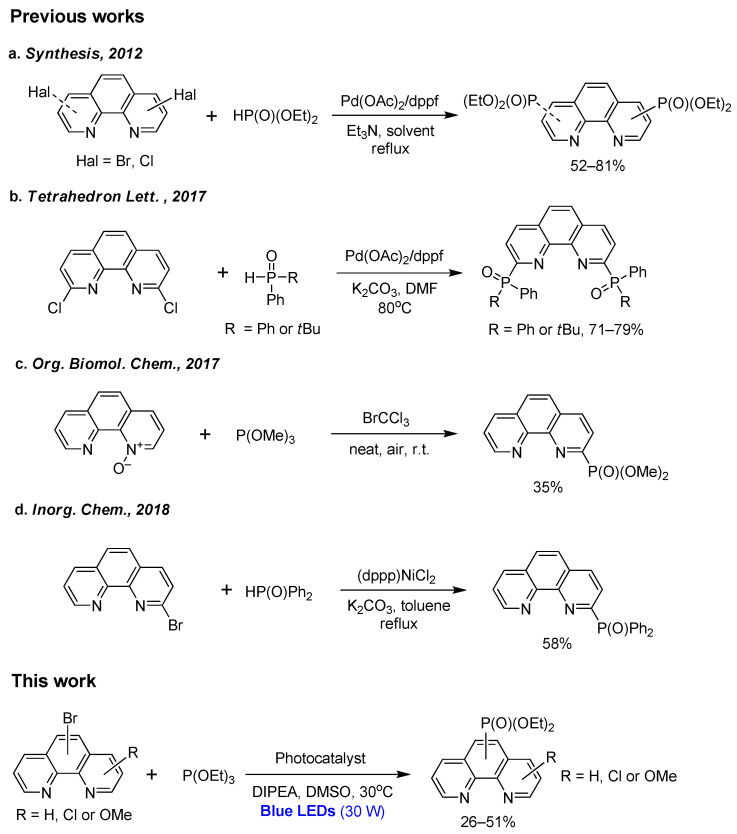
Synthetic approaches to phosphonate-substituted 1,10-phenanthrolines [11,16,25,26].

The introduction of phosphonate groups into the ligand is of considerable interest since these substituents affect the electronic properties of the complexes, help to increase the solubility of the ligand and complexes in polar media [12], and can also be used as anchor groups for the immobilization of molecules on solid oxide substrates [13] or into the structure of coordination polymers (MOFs) to create hybrid materials with valuable properties [14]. A universal method for the synthesis of phosphonate-substituted 1,10-phenanthrolines is Pd-catalyzed phosphonylation (Hirao reaction [15]), which allows a wide range of substituted ligands to be obtained (Scheme 1a,b) [11,16], which have found application as components of heterogeneous catalysts [17,18], photocatalysts [19,20], and reagents for the extractive separation of *f*-elements [21,22,23,24]. Specific cases of the synthesis of 2-phosphonate-substituted 1,10-phenanthrolines using a Ni-catalyst (Scheme 1d) [25] or a reaction with 1,10-phenanthroline *N*-oxide (Scheme 1c) [26] were also reported.

In recent years, methods using visible light photoredox catalysis have been substantially developed [27,28,29,30]. This approach allows the generation of radical intermediates under mild conditions, which helped to make a number of transformations of this type more energy efficient and gave way to many new, previously unknown reactions [31,32,33]. Thus, many different approaches have been proposed for the phosphonylation of various aromatic substrates [34,35,36,37,38,39]. Among other reactions, various approaches have been reported for the formation of the C(sp^2^)–P bond using halogenated arenes in the nickel/photoredox-catalyzed coupling reaction under visible light (dual catalysis) [40,41,42]. In 2016, König et al. [43] proposed a protocol for the phosphonylation of bromoarenes using triethyl phosphite in the presence of an organic sensitizer (Rhodamine 6G) under visible light which was further adjusted for other substrates in subsequent studies [44,45,46,47,48,49,50,51,52,53]. These methods are based on the photoinduced generation of an aryl radical, which then reacts with the phosphite to form a C–P bond. An important advantage of such reactions is their transition metal-free character, which makes them attractive for the functionalization of substrates that can act as chelators of transition metals. In this work, we investigated the possibilities and limitations of the visible light photoredox phosphonylation reaction using triethyl phosphite for the synthesis of phosphonate-substituted 1,10-phenanthrolines.

## 2. Results and Discussion

**Figure 1:**
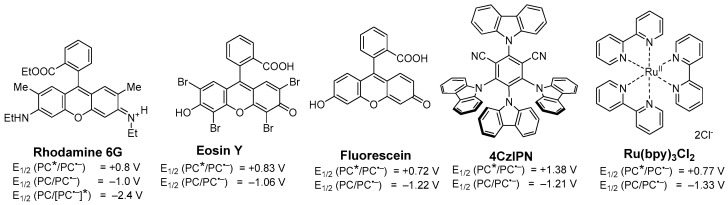
Photocatalysts studied in this work and their redox potentials (vs. SCE) in the ground and excited states [54,55].

The reaction of 5-bromo-1,10-phenanthroline (**1**) with triethyl phosphite was chosen as a model (Table 1). The process was carried out in dry DMSO in the presence of a three-fold excess of triethyl phosphite and a two-fold excess of DIPEA, which simultaneously acted as a base and a reducing agent. The reaction mixture was degassed before irradiation, and the reaction proceeded under dry argon. The studied photocatalysts are shown in Figure 1.

The reaction mixture was analyzed with ^1^H NMR spectroscopy using an internal standard. Initially, Rhodamine 6G, which is known to readily generate aryl radicals due to the conPET process, was studied as a photocatalyst [56]. When the reaction mixture was irradiated with green light (12 W, 525 nm) at room temperature for 16 h, no conversion of starting compounds was observed. After the irradiation with blue light of similar power (12 W, 455 nm), the reaction also did not proceed (entries 1, 2). Increasing the irradiation power to 30 W led to only a low conversion of the starting phenanthroline **1** (entry 3). The formation of target product **2** in 13% yield was observed in the reaction mixture, and the product of the C–Br bond reduction (Phen, 4% yield) was also observed. It should be noted that the use of a more powerful irradiation source led to heating of the reaction mixture up to 30 °C. The use of 1,2,3,5-Tetrakis(carbazol-9-yl)-4,6-dicyanobenzene (4CzIPN) as a photocatalyst led to a 50% conversion of the initial bromophenanthroline and the target product was formed in 27% yield after 22 h (entry 4). In this case, the complete degradation of the photocatalyst could be observed visually. An additional portion of the photocatalyst and further irradiation for 16 h provided only a slight increase in the conversion and the product yield (entry 5). The use of Ru(bpy)_3_^2+^ as a photocatalyst, which is characterized by higher photostability, helped to achieve somewhat better conversion and yield after 16 h of irradiation (entry 6). The highest yield of the phosphonylation product **2** after 16 h of irradiation was observed when using the Eosin Y dye (entry 7). Longer irradiation of the reaction mixture for an additional 22 h and introduction of a new portion of the photocatalyst resulted in a high conversion of phenanthroline **1** and provided a 52% yield of product **2**. In this case, the formation of the reduction product (Phen) was also observed, with a yield of 16%. The use of Fluorescein as a photocatalyst also ensured high conversion of the initial bromophenanthroline, but the yield of the phosphonylation product was poorer (entries 9, 10). We have shown that increasing the Eosin Y loading to 15 mol% at the beginning of the reaction led to a high conversion after 22 h and retained the yield of the phosphonylation product (entry 11). Using other solvents commonly employed in such reactions demonstrated that DMSO is the best choice (entries 12, 13).

**Scheme 2:**
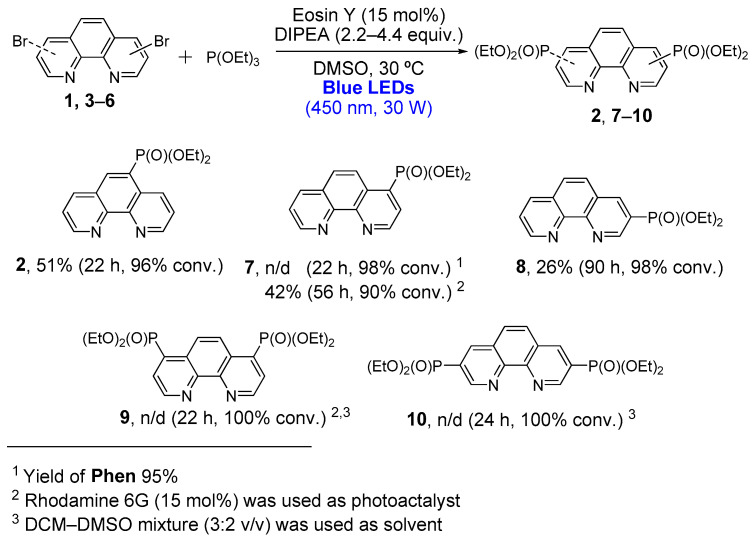
Photoredox-catalyzed phosphonylation of bromo- and dibromo-substituted 1,10-phenanthrolines. Reaction conditions: bromo-1,10-phenanthroline (0.375 mmol), Eosin Y (15 mol%), DMSO (2 mL), P(OEt)_3_ (3–6 equiv.), DIPEA (2.2–4.4 equiv.), 30 W blue LEDs (450 nm), 30 °C, argon atmosphere. The yield and conversion were determined with NMR ^1^H analysis of reaction mixtures, 1,4-diacetoxybenzene was used as an internal standard.

Next, we investigated the possibility of phosphonylation of other bromo-substituted phenanthrolines under optimized conditions (Scheme 2). It was found that the position of the bromine atom in the heterocyclic ring dramatically changes the reactivity of the substrate. The reaction of 4-bromo-1,10-phenanthroline **3** did not lead to the phosphorylation product **7**, and after 22 h, the formation of the reduction product Phen was observed in 95% yield. By changing the photocatalyst for Rhodamine 6G, we managed to obtain target product **7** with a 42% yield. However, the reaction time had to be increased to 56 h. 3-Bromo-1,10-phenanthroline **4** under optimized conditions was much less reactive, and its high conversion was achieved only after 90 h of irradiation, while the yield of target product **8** was only 26%.

4,7-Dibromo-1,10-phenanthroline (**5**) and 3,8-dibromo-1,10-phenanthroline (**6**) are practically insoluble in DMSO; thus, the reaction was carried out upon the addition of dichloromethane. Despite the complete conversion of the initial dibromophenanthrolines, neither the target phosphonylation products **9** and **10** nor the reduction products could be detected in the reaction mixture, which is most probably due to the formation of polymers and the destruction of the heterocyclic system under the reaction conditions.

**Figure 2:**
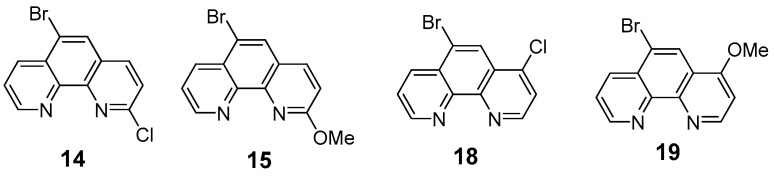
Functionalized 1,10-phenanthrolines.

Afterwards, we studied the effect of other substituents in 1,10-phenanthroline on the process. For this purpose, we synthesized functionalized analogs of compound **2** (Figure 2) containing electron-donor and electron-withdrawing substituents at positions 2 and 4 of the 1,10-phenanthroline core.

**Scheme 3:**
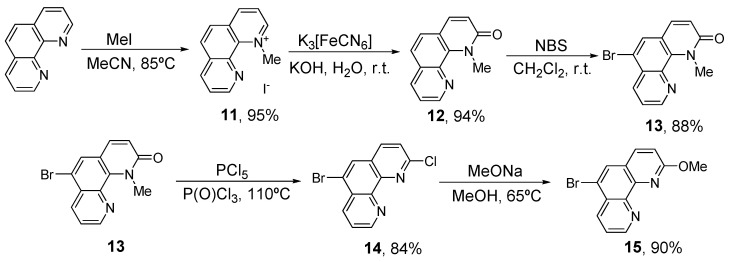
Synthesis of compounds **14** and **15**.

Compound **14** was synthesized in four steps starting from 1,10-phenanthroline (Scheme 3). The method is based on the classical approach to 2-chloro-1,10-phenanthroline [57]. We found that 1-methyl-1,10-phenanthrolin-2(1H)-one selectively forms bromo-substituted product **13** in high yield in the reaction with NBS in dichloromethane at room temperature. Chlorination-deoxygenation of compound **13** under standard conditions gave the target 6-bromo-2-chloro-1,10-phenanthroline **14** in a good yield. The reaction of compound **14** with sodium methylate selectively provided 2-methoxy-substituted 1,10-phenanthroline **15** by aromatic nucleophilic substitution reaction.

**Scheme 4:**
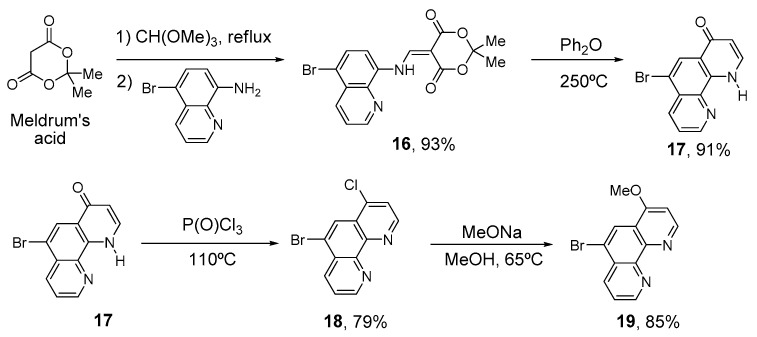
Synthesis of compounds **18** and **19**.

Phenanthroline **18** was obtained in three steps (Scheme 4). At the first step, Meldrum’s acid, *ortho*-methyl formate, and 8-amino-5-bromoquinoline were condensed to form adduct **16**, which at the next step underwent cyclization upon heating in diphenyl ether to give 6-bromo-1,10-phenanthrolin-4(1H)-one (**17**). Refluxing this compound in P(O)Cl_3_ afforded the target 6-bromo-4-chloro-1,10-phenanthroline (**18**). Treatment of the compound **18** with sodium methylate gave 6-bromo-4-methoxy-1,10-phenanthroline (**19**) in a good yield.

**Scheme 5:**
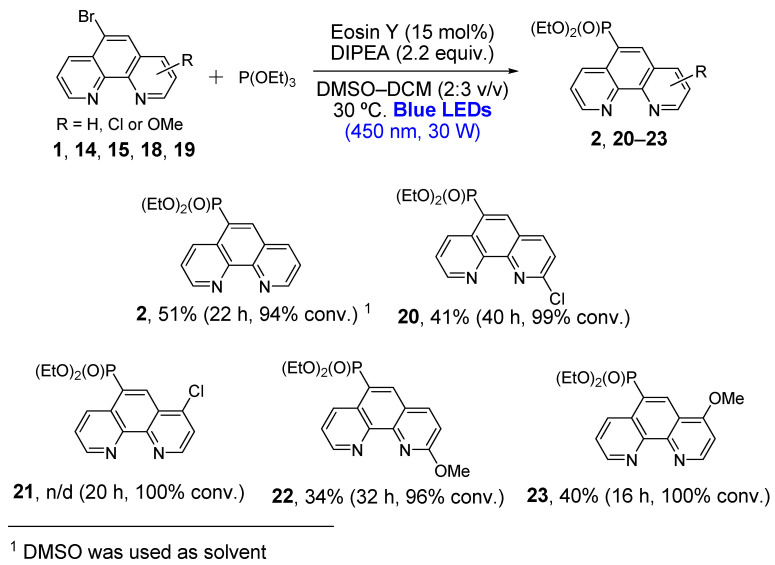
Photoredox-catalyzed phosphonylation of functionalized 1,10-phenanthrolines. Reaction conditions: bromo-1,10-phenanthroline (0.375 mmol), Eosin Y (15 mol%), DMSO (2 mL), DCM (3 mL), P(OEt)_3_ (3 equiv.), DIPEA (2.2 equiv.), 30 W blue LEDs (450 nm), 30 °C, argon atmosphere. The yield and conversion were determined with NMR ^1^H analysis of reaction mixtures, 1,4-diacetoxybenzene was used as an internal standard.

Then, the obtained compounds were introduced in the photoredox-catalyzed phosphonylation reaction (Scheme 5). As disubstituted 1,10-phenanthrolines **14**, **15**, **18**, and **19** are poorly soluble in DMSO, the reaction was carried out in a DMSO–DCM mixture, which ensured the dissolution of the starting compounds. The reaction was monitored by ^1^H and ^31^P NMR spectroscopy. The reaction mixtures were irradiated until the conversion of the initial bromophenanthroline surpassed 90%. Different reactivity was observed for these compounds. In the case of 6-bromo-2-chloro-1,10-phenanthroline (**14**), complete conversion was observed after 40 h, and selective substitution of the bromine atom took place, while the yield of target product **20** was 41%. It should be noted that such substitution selectivity is quite difficult to achieve using classical Pd-catalyzed phosphonylation, since the chlorine atom at position 2 is also readily substituted under the reaction conditions, leading to the formation of a disubstituted product [8,11]. Isomeric 6-bromo-4-chloro-1,10-phenanthroline (**18**) turned out to be more labile, and complete conversion of the substrate was observed after 20 h. However, this compound reacted similarly to dibromophenanthrolines **9** and **10**, and neither a phosphonate nor reduction product could be observed.

In the case of a methoxy-substituent at position 2 of the phenanthroline ring, the yield of target product **22** was 34%. Compound **19** turned out to be the most reactive; complete conversion was achieved after 16 h of irradiation of the reaction mixture, and the yield of the target phosphonylation product was 40%. It is important that in all cases, the formation of the product of C–Br bond reduction was also observed in yields of 13–16%.

**Scheme 6:**
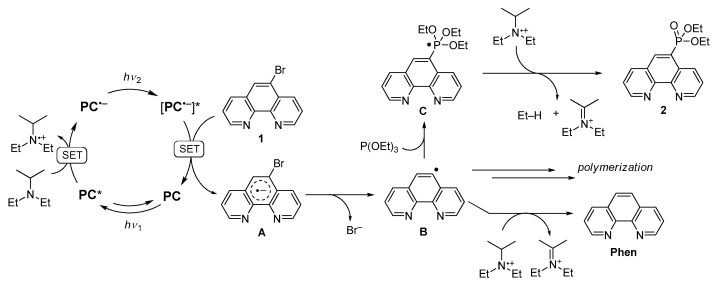
Proposed mechanism of photoredox-catalyzed phosphonylation of 1,10-phenanthrolines.

The following reaction mechanism is proposed on the basis of literature data (Scheme 6) [43,47,51]. The excited photocatalyst PC* oxidizes DIPEA (+0.68 V vs. SCE [58]) to form radical anion PC^•–^, which is then excited to [PC^•–^]*. This excited anion reduces bromo-substituted 1,10-phenanthroline to corresponding radical anion **A** (*via* single electron transfer), which then decomposes, giving Br^–^ anion and radical **B [59]**. This radical interacts with triethylphosphine, giving phosphoranyl radical **C**, which then loses reactive ethyl radical, forming target phosphonylation product **2**. The reduction potential for 5-bromo-1,10-phenanthroline **1** was reported to be −1.75 V vs. SCE [59], which means that PC^•–^ has insufficient reducing potential (for each studied catalysts, see Figure 1) and participation of [PC^•–^]* is to be proposed.

**Figure 3:**
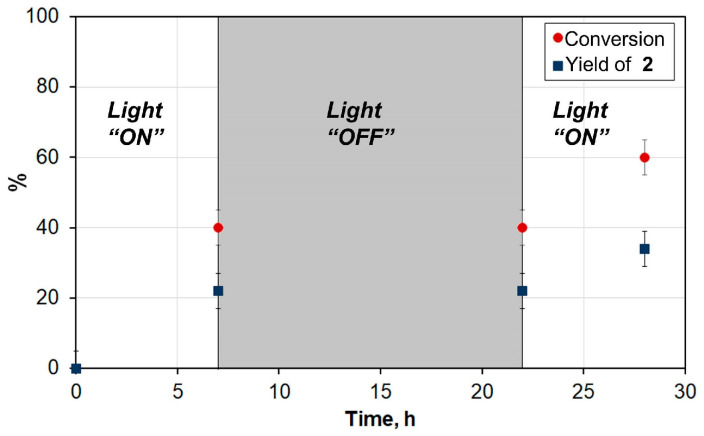
NMR-monitoring of the model reaction under ON/OFF irradiation. Reaction conditions: 5-bromo-1,10-phenanthroline **1** (0.375 mmol), Eosin Y (15 mol%), DMSO (2 mL), P(OEt)_3_ (3 equiv.), DIPEA (2.2 equiv.), 30 W blue LEDs (450nm), 30 °C, argon atmosphere. The yield and conversion were determined with NMR ^1^H analysis of reaction mixtures. 1,4-diacetoxybenzene was used as a internal standard.

The standard control experiments were conducted the results of which were in agreement with the proposed mechanism (see Table S1 in the Supporting Information). The conversion and the yield of the product decreased significantly in the presence of 1.5 equiv. of radical trap (TEMPO) while the addition of 4 equiv. of TEMPO totally suppressed the reaction. The heteroaryl radical intermediate was detected by ESI-MS, which supports a radical reaction mechanism (Figure S3). No conversion was observed in the absence of DIPEA, which indicates that this sacrificial reductant is necessary for the process. The reaction does not proceed without irradiation. Neither the formation of product **2** nor the conversion of **1** was observed in the dark phase of the ON/OFF irradiation experiment (Figure 3), which excludes any significant contribution from the radical chain mechanism and supports the visible light promoted pathway. The TON values were calculated to be 2–4 depending on the substrate (Table S2), which confirms the catalytic nature of the reaction. The formation of a small amount of the product was observed under irradiation in the absence of the photocatalyst, which is apparently due to the ability of the starting phenanthroline to act also as photosensitizer and give intermediate **A**. In the case of 5-bromo- and 4-bromo-1,10-phenanthrolines (**1** and **3**), the formation of phosphonylation (8–12%) and reduction (8–24%) products was observed in the mixture, but in the case of 3-bromo-1,10-phenanthroline **4** only decomposition of the starting compound was observed. Thus, photocatatalyst-independent photochemical processes also contribute to both the formation of the phosphonylation product and side reactions. It should also be noted that the other mechanisms of this reaction were also proposed [44,60], and they can not totally be excluded.

The high irradiation power required for the reaction to proceed, as well as the long reaction time, indicate a very low quantum yield. This can be explained by the reverse oxidation of the unstable anion-radical **A** back to the starting bromophenanthroline. 1,10-Phenanthroline radical **B** also tends to polymerize under reduction conditions [61,62], which seems to be the main side reaction in our case. The radical **B** can also intercept hydrogen and give 1,10-phenanthroline. The position of the halogen in the heterocycle has no significant influence on the reduction potential of the halogenated heterocycles (e.g., pyridines [63], pyrimidines [64], quinolines [47]), but the presence of the additional substituents can have a significant effect. We suppose that a different reactivity is mostly due to the different formation rates and reactivity of the corresponding heteroaryl radicals **B**. If the reactivity of the radical is high or the radical is being formed too fast, then the contribution of side reactions should increase leading to decrease of the product yield. This leads to low TON values (ca. 2–4), taking into account the high photocatalyst load. Due to these facts, careful optimization of the reaction conditions is required for each substrate to achieve acceptable yields.

**Scheme 7:**
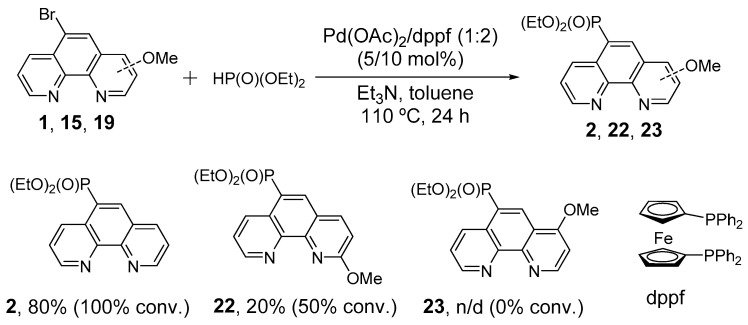
Pd-catalyzed phosphonylation of methoxy-substituted 1,10-phenanthrolines.

We also investigated an alternative route to methoxy-substituted phosphonate-containing 1,10-phenanthrolines using a previously developed palladium-catalyzed phosphonylation reaction (Scheme 7). The reaction was carried out under reflux in toluene in the presence of the Pd(OAc)_2_/dppf (5/10 mol%) catalytic system and triethylamine as a base.

In the absence of additional substituents in 1,10-phenanthroline, target product **2** is formed in high yield. The presence of a methoxy group at position 2 decreases the reactivity of the substrate substantially, as well as the selectivity of the reaction. Target product **22** was obtained with only a 20% yield and a 50% conversion of the starting compound **15** in 24 h. In the case of compound **19** with the methoxy group at position 4, no conversion was observed even after 24 h. The observed phenomenon can be explained as follows. The introduction of an electron-donor group at *ortho*- or *para*- position to the heterocyclic nitrogen atom in 1,10-phenanthroline increases its ability to form a complex with palladium, which hampers the reaction. In the case of compound **15**, this effect is apparently partially balanced by the steric hindrances created by the methoxy group at the coordinating site, thus allowing the reaction to proceed. Compound **19** is more prone to bind palladium due to the lack of sterical hindrance, which completely prevents the reaction from proceeding. As a result, product **23** could only be prepared by a metal-free photoredox-catalyzed reaction.

## 3. Materials and Methods

### 3.1. Reagents

Unless otherwise noted, all chemicals and starting materials were obtained commercially from Alfa Aesar (Thermo Fisher Scientific Co., Ward Hill, MA, USA), J&K Scientific (San Jose, CA, USA), and Aldrich–Sigma (Merck Co., Rahway, NJ, USA) and used without further purification. Preparative column chromatography was carried out using Machery Nagel Silica gel 60 (40⎼63 µm) from Merck Co. (Rahway, NJ, USA). CH_2_Cl_2_ and MeCN were distilled over CaH_2_. DMSO and DMF were distilled over CaH_2_ under argon flow and stored over 3Ǻ-sieves under argon. MeOH was used freshly distilled.

### 3.2. Apparatus

^1^H, ^13^C and ^31^P NMR spectra were recorded with a Bruker Avance-400 spectrometer (Bruker Daltonics, Bremen, Germany) in CDCl_3_, CD_3_OD, or DMSO-*d*_6_ using the residual signal of CHCl_3_, CD_2_HOD, or DMSO-HD_5_ as internal standards. HRMS ESI mass-spectra were obtained on a TripleTOF 5600+ mass-spectrometer (AB Sciex, Framingham, MA, USA) using the following parameters: a voltage of 5.5 kV for positive ions mode, 4.5 kV for negative ions mode; ion source gas 15 arb, curtain gas 20 arb; eluent MeCM + 0.1% HCOOH. MALDI-TOF mass-spectra were registered on a Bruker Daltonics Autoflex II mass-spectrometer (Bruker Daltonics, Bremen, Germany) in positive ion mode with a dithranol matrix and polyethyleneglycols as internal standards. FTIR spectra were registered on a Nicolet iS 5 (Thermo Fisher Scientific, Madison, WI, USA) with iD3 ATR accessory (ZnSe). Photocatalyzed reactions were carried out using a PhotoRedOx Box photoreactor (HepatoChem, Beverly, NJ, USA) (see Figure S1 in the Supporting Information).

### 3.3. Synthesis of Starting Compounds

5-Bromo-1,10-phenathroline (**1**) was obtained from 1,10-phenanthroline using the previously reported procedure [65]. 3-Bromo-1,10-phenanthroline (**4**) and 3,8-dibromo-1,10-phenanthroline (**6**) were synthesized using bromination of 1,10-phenanthroline hydrochloride [66]. 4-Bromo-1,10-phenanthroline (**3**) [67,68] and 4,7-dibromo-1,10-phenanthroline (**7**) [69] were prepared using known three-step procedures from 8-aminoquinoline and *o*-phenylenediamine, respectively. 5-Bromoquinoline-8-amine was synthesized according to the previously reported procedure [70].

*1-Methyl-1,10-phenanthrolinium iodide (***11***)* [57]. A round-bottom flask equipped with a condenser and magnetic stirrer was charged with 1,10-phenanthroline monohydrate (11 g, 55 mmol) and acetonitrile (50 mL), then iodomethane (10.5 mL, 3 equiv.) was added, and the mixture was stirred at reflux for 1.5 h. After cooling, the yellow precipitate formed was collected by filtration, washed with ethanol, and dried under reduced pressure. Yield 16.8 g (95%), yellow powder. ^1^H NMR (400 MHz, CDCl_3_) δ 9.58 (d, 1H, *J* = 5.8 Hz), 9.38 (d, 1H, *J* = 8.1 Hz), 9.25–9.28 (m, 1H), 8.75 (d, 1H, *J* = 8.7 Hz), 8.32–8.44 (m, 3H), 8.02 (dd, 1H, *J* = 8.1, 4.3 Hz), 5.24 (s, 3H, CH_3_).
*1-Methyl-1,10-phenanthrolin-2{1H}-one (***12***)* [57]. Suspension of **11** (16.6 g, 51 mmol) in water (310 mL) and solution of NaOH (59.9 g in 310 mL of water) were simultaneously added drop by drop into the stirred solution of K_3_[Fe(CN)_6_] (42.8 g, 0.13 mol) in 310 mL at 5 °C in ice bath during 30 min. The solution was additionally stirred for 1.5 h. The beige precipitate was collected by filtration and washed with water. The precipitate was dissolved in CH_2_Cl_2_ (ca. 400 mL), and the solution was dried over Na_2_SO_4_ and concentrated in vacuo. Yield 10.2 g (94%), beige solid. ^1^H NMR (400 MHz, CDCl_3_) δ 8.95 (dd, *J* = 4.1, 1.8 Hz), 8.18 (dd, 1H, *J* = 8.2, 1.8 Hz), 7.79 (d, 1H, *J* = 9.3 Hz), 7.58 (d, 1H, *J* = 8.4 Hz), 7.55 (d, 1H, *J* = 8.4 Hz), 7.50 (dd, 1H, *J* = 8.2, 4.1 Hz), 6.91 (d, 1H, *J* = 9.3 Hz), 4.49 (s, 3H, CH_3_).
*6-Bromo-1-methyl-1,10-phenanthrolin-2{1H}-one (***13***)*. 1-Methyl-1,10-phenanthrolin-2-one **12**(2.73 g, 13 mmol) was dissolved in 30 mL of CH_2_Cl_2_ in a 100 mL flask, *N*-bromosuccinimide (2.55 g, 14.3 mmol) was then added and the resulting solution was stirred at r.t. for 4 h. Then, the solution was washed with water, dried over Na_2_SO_4_, and concentrated under reduced pressure. Yield 3.31 g (88%), pale yellow solid. ^1^H NMR (400 MHz, CDCl_3_) δ 8.97 (dd, ^3^*J* = 4.1, ^4^*J* = 1.7 Hz, 1H), 8.62 (dd, ^3^*J* = 8.5, ^4^*J* = 1.7 Hz, 1H), 7.89 (s, 1H), 7.72 (d, ^3^*J* = 9.3 Hz, 1H), 7.62 (dd, ^3^*J* = 8.5, ^3^*J* = 4.1 Hz, 1H), 6.94 (d, ^3^*J* = 9.3 Hz, 1H), 4.44 (s, 3H); ^13^C{^1^H} NMR (100.6 MHz, CDCl_3_) δ 164.0, 147.6, 140.7, 138.1, 137.7, 135.6, 129.8, 128.6, 123.0, 122.9, 120.8, 114.9, 38.1. IR (neat, ν_max_, cm^−1^): 610, 654, 782, 794, 811, 821, 833, 846, 878, 994, 1034, 1134, 1163, 1188, 1204, 1291, 1308, 1361, 1381, 1415, 1457, 1592, 1612, 1666 (C=O). MALDI-TOF: *m*/*z* found 288.9950. C_13_H_10_BrN_2_O^+^. Calcd. 288.9971 [M + H]^+^.
*6-Bromo-2-chloro-1,10-phenanthroline (***14***)*. An oven-dried 50 mL flask was charged with 6-bromo-1-methyl-1,10-phenanthrolin-2{1H}-one **13** (3 g, 10.4 mmol), PCl_5_ (2.69 g, 12.9 mmol), and POCl_3_ (18 mL) under a N_2_ atmosphere. The flask was fitted with an oven-dried reflux condenser, and the mixture was refluxed overnight. The POCl_3_ excess was removed under reduced pressure. Cold water (50 mL) was then added to the residual solid, and the mixture was neutralized with ammonia solution. The obtained suspension was extracted with CH_2_Cl_2_ (3 × 30 mL). The combined organic layers were dried over Na_2_SO_4_, and the solvent was concentrated under reduced pressure. Yield 2.56 g (84%), white solid. ^1^H NMR (400 MHz, CDCl_3_) δ 9.30 (dd, ^3^*J* = 4.4, ^4^*J* = 1.6 Hz, 1H), 8.72 (dd, ^3^*J* = 8.4, ^4^*J* = 1.6 Hz, 1H), 8.15 (s, 1H), 8.14 (d, ^3^*J* = 8.4 Hz, 1H), 7.82 (dd, ^3^*J* = 8.4, ^3^*J* = 4.4 Hz, 1H), 7.65 (d, ^3^*J* = 8.4 Hz, 1H); ^13^C{^1^H} NMR (100.6 MHz, CDCl_3_) δ 151.8, 151.3, 145.34, 145.31, 137.8, 135.9, 128.8, 128.2, 127.36, 125.0, 124.3, 121.2. IR (neat, ν_max_, cm^−1^): 619, 641, 668, 692, 714, 738, 757, 805, 819, 861, 871, 882, 916, 965, 1036, 1069, 1086, 1138, 1192, 1279, 1288, 1310, 1375, 1398, 1438, 1473, 1490, 1549, 1580, 1594. MALDI-TOF: *m*/*z* found 292.9500. C_12_H_7_BrClN_2_^+^. Calcd. 292.9476 [M + H]^+^.
*5-((5-Bromoquinolin-8-yl)imino)-2,2-dimethyl-1,3-dioxane-4,6-dione (***16***)*. A 100 mL two-neck flask equipped with a magnetic stirrer and condenser was charged with Meldrum’s acid (3.67 g, 25 mmol) and CH(OMe)_3_ (17 mL). The mixture was stirred under reflux for 1 h. Then, 5-bromoquinolin-8-amine (3.81 g, 17 mmol) was added to the hot mixture, and the mixture was additionally refluxed for 1 h. Then, the reaction mixture was cooled, the precipitate was collected, washed with diethyl ether (3 × 10 mL), and dried in air. Yield 5.97 g (93%), light-brown solid. M.p. 200–201 °C (decomp.). ^1^H NMR (CDCl_3_, 400 MHz) δ 8.87 (dd, 1H, *^3^J* = 4.2 Hz, *^4^J* = 1.4 Hz, 2-H (Quin)), 8.77 (s, 1H, CHNH), 8.42 (dd, 1H, *^3^J* = 8.6, *^4^J* = 1.4 Hz, 4-H (Quin)), 7.74 (d, 1H, *^3^J* = 8.3 Hz, 6-H (Quin)), 7.53 (dd, 1H, *^3^J* = 8.6, *^4^J* = 4.2 Hz, 3-H (Quin)), 7.50 (d, 1H, *^3^J* = 8.4 Hz, 7-H (Quin)), 3.72 (s, 1H, NH), 1.66 (s, 6H, CH_3_). ^13^C{^1^H} NMR (CDCl_3_, 100.6 MHz) δ 164.7 (1C, C=O), 164.1 (1C, C=O), 150.4 (1C), 149.7 (1C), 138.9 (1C), 135.6 (1C), 133.9 (1C), 129.9 (1C), 127.7 (1C), 123.5 (1C), 117.9 (1C), 112.4 (1C), 105.1 (1C), 88.4 (1C), 26.7 (2C, CH_3_). IR (neat, ν_max_, cm^−1^): 632, 639, 651, 688, 729, 749, 789, 811, 826, 833, 852, 887, 913, 937, 991, 1005, 1017, 1031, 1051, 1119, 1140, 1204 (C-N), 1221, 1261 (C-N), 1281 (C-N), 1311, 1356, 1370, 1381, 1392, 1443, 1471, 1537, 1591 (C=N), 1621, 1650, 1682, 1727 (C=O), 2359. ESI-TOF: *m*/*z* found 377.0128. C_16_H_13_BrN_2_O_4_. Calcd. 377.0137 [M + H]^+^.
*6-Bromo-1,10-phenanthrolin-4{1H}-one (***17***)*. Compound **16** (5.97 g, 15.8 mmol) was slowly added into a 250 mL flask with boiling diphenyl ether (60 mL) stirred at ca. 245 °C. The mixture was additionally stirred under reflux for 1 h. When the mixture temperature dropped to 60 °C, hexanes (60 mL) were added, and the mixture was cooled to room temperature. The precipitate was collected and washed with hexanes (2 × 20 mL) and diethyl ether (3 × 20 mL), then dried under reduced pressure. Yield 3.99 g (91%), fine brownish powder. M.p. 206–207 °C. ^1^H NMR (DMSO-*d*_6_, 400 MHz) δ 12.6 (s, 1H, NH), 9.06 (d, 1H, *^3^J* = 4.2 Hz, 9-H), 8.53 (d, 1H, *^3^J* = 8.4 Hz, 7-H), 8.30 (s, 1H, 5-H), 7.95 (d, 1H, *^3^J* = 7.3 Hz, 2-H), 7.89 (dd, 1H, *^3^J* = 8.4, *^4^J* = 4.2 Hz, 8-H), 6.34 (d, 1H, *^3^J* = 7.3 Hz, 3-H). ^13^C{^1^H} NMR (DMSO-*d*_6_, 100.6 MHz) δ 175.0 (1C, C=O), 150.2 (1C), 139.7 (1C), 139.3 (1C), 136.9 (1C), 135.6 (1C), 127.5 (1C), 125.6 (1C), 125.3 (1C), 124.2 (1C), 115.0 (1C), 112.3 (1C). IR (neat, ν_max_, cm^−1^): 649, 669, 720, 731, 761, 823, 998, 1023, 1049, 1152, 1293(C-N), 1507, 1521, 1540, 1559, 1617, 1636, 1647, 1653 (C=O), 1684, 1700. ESI-TOF: *m*/*z* found 274.9818. C_12_H_8_BrN_2_O. Calcd. 274.9820 [M + H]^+^.
*6-Bromo-4-chloro-1,10-phenanthroline (***18***)*. A 100 mL flask equipped with a condenser and magnetic stirrer was charged with 6-bromo-1,10-phenanthrolin-4{1H}-one **17** (2.5 g, 9.1 mmol) and POCl_3_ (23 mL). The mixture was stirred under reflux for 3 h. The POCl_3_ excess was removed under reduced pressure. Cold water (50 mL) was then added to the residual solid, and the mixture was neutralized with ammonia solution until it reached a pH of 8. The product was extracted with CH_2_Cl_2_ (3 × 30 mL). Combined organic layers were dried over Na_2_SO_4_ and concentrated under reduced pressure. Yield 2.12 g (79%), light gray solid. M.p. 204–205 °C. ^1^H NMR (CDCl_3_, 400 MHz) δ 9.10 (dd, 1H, *^3^J* = 4.3 Hz, *^4^J* = 1.6 Hz, 9-H), 8.93 (d, 1H, *^3^J* = 4.8 Hz, 2-H), 8.57 (dd, 1H, *^3^J* = 8.4 Hz, *^4^J* = 1.6 Hz, 7-H), 8.44 (s, 1H, 5-H), 7.70 (dd, 1H, *^3^J* = 8.4 Hz, *^3^J* = 4.3 Hz, 8-H), 7.64 (d, 1H, *^3^J* = 4.8 Hz, 3-H). ^13^C{^1^H} NMR (DMSO-*d*_6_, 100.6 MHz): δ 151.0 (1C), 149.8 (1C), 146.2 (1C), 145.7 (1C), 141.5 (1C), 136.0 (1C), 127.9 (1C), 126.7 (1C), 125.3 (1C), 124.3 (1C), 123.8 (1C), 122.3 (1C). IR (neat, ν_max_, cm^−1^): 605, 611, 676, 691, 725, 741, 795, 816, 837, 876, 920, 1042, 1052, 1125, 1192, 1240, 1271, 1278, 1337, 1386, 1408, 1435, 1472, 1498, 1551 (C=N), 1575 (C=N), 1592 (C=N). ESI-TOF: *m*/*z* found 292.9480. C_12_H_7_BrClN_2_. Calcd. 292.9481 [M + H]^+^.
*6-Bromo-2-methoxy-1,10-phenanthroline (***15***)*. A 10 mL flask equipped with a condenser and magnetic stirrer was charged with dry MeOH (3.6 mL), and metallic sodium (28 mg, 1.2 mmol) was added. After the complete dissolution of sodium, 6-bromo-2-chloro-1,10-phenanthroline (**14**) (294 mg, 1 mmol) was added, and the mixture was stirred under reflux for 24 h. Then, the mixture was allowed to cool, water (20 mL) was added, and the product was extracted in CH_2_Cl_2_ (3 × 10 mL). Organic layers were combined, dried over Na_2_SO_4_, and concentrated under reduced pressure. Yield 261 mg (90%), brownish solid. M.p. 156–158 °C. ^1^H NMR (CDCl_3_, 400 MHz): δ 9.11 (d, 1H, *^3^J* = 4.2 Hz, 9-H), 8.52 (d, 1H, *^3^J* = 8.4 Hz, 7-H), 7.91 (s, 1H, 5-H), 7.88 (d, 1H, *^3^J* = 8.7 Hz, 4-H), 7.59 (dd, 1H, *^3^J* = 8.4, *^3^J* = 4.2 Hz, 8-H), 7.01 (d, 1H, *^3^J* = 8.7 Hz, 3-H), 4.24 (s, 3H, CH_3_). ^13^C{^1^H} NMR (DMSO-*d*_6_, 100.6 MHz): δ 163.2 (1C), 150.3 (1C), 145.5 (1C), 143.7 (1C), 137.8 (1C), 135.7 (1C), 129.2 (1C), 127.8 (1C), 124.8 (1C), 123.0 (1C), 117.0 (1C), 114.4 (1C), 53.9 (1C, CH_3_). IR (neat, ν_max_, cm^−1^): 618, 625, 656, 669, 691, 720, 748, 798, 814, 823, 871, 922, 1013, 1137 (C-O-C), 1225, 1270, 1300, 1354, 1375, 1399, 1427, 1464, 1493, 1558 (C=N), 1590 (C=N), 1612. ESI-TOF: *m*/*z* found 288.9974. C_13_H_10_BrN_2_O. Calcd. 288.9977 [M + H]^+^.
*6-Bromo-4-methoxy-1,10-phenanthroline (***19***)* was synthesized using a procedure similar to the compound **15** from 6-bromo-4-chloro-1,10-phenanthroline **18** (294 mg, 1 mmol) and sodium (28 mg, 1.2 mmol) in methanol (3.4 mL). Yield 245 mg (85%), brownish solid. M.p. 219–220 °C. ^1^H NMR (CDCl_3_, 400 MHz): δ 9.12 (d, 1H, *^3^J* = 4.2 Hz, 9-H), 8.95 (d, 1H, *^3^J* = 5.2 Hz, 2-H), 8.57 (d, 1H, *^3^J* = 8.3 Hz, 7-H), 8.45 (s, 1H, 5-H), 7.64 (dd, 1H, *^3^J* = 8.3, *^3^J* = 4.2 Hz, 8-H), 6.93 (d, 1H, *^3^J* = 5.2 Hz, 3-H), 4.00 (s, 3H, CH_3_). ^13^C{^1^H} NMR (DMSO-*d*_6_, 100.6 MHz): δ 161.2 (1C), 151.6 (1C), 150.6 (1C), 146.3 (1C), 135.7 (1C), 127.8 (1C), 123.7 (1C), 123.7 (1C), 123.6 (1C), 120.8 (1C), 119.4 (1C), 103.4 (1C), 55.9 (1C, CH_3_). 625, 650, 669, 678, 697, 750, 804, 819, 832, 847, 878, 921, 1029, 1052, 1085(C-O-C), 1149, 1282, 1307, 1343, 1389, 1410, 1437, 1451, 1489, 1507, 1558 (C=N), 1587 (C=N), 1601 (C=N), 2332. ESI-TOF: *m*/*z* found 288.9974. C_13_H_10_BrN_2_O. Calcd. 288.9977 [M + H]^+^.

### 3.4. Photoredox-Catalyzed Phosphonylation of Bromophenanthrolines

*General procedure.* Bromo-substituted 1,10-phenanthroline (0.375 mmol), Eosin Y (36 mg, 0.056 mmol, 15 mol.%), and a magnetic stir bar were placed into a Schlenk tube, and the vessel was filled with argon. Then, DMSO (2 mL), triethyl phosphite (195 μL, 3 equiv.), and DIPEA (150 μL per bromine, 2.2 equiv.) were added in a stream of argon. The mixture was degassed *via* sonication under reduced pressure after the addition of each reagent. For the synthesis of compounds **20**–**23**, degassed CH_2_Cl_2_ (3 mL) was added in the argon flow after the addition of other reagents to provide dissolution of the substrate. The mixture was stirred under blue LEDs irradiation (30 W, 450 nm) in a photoreactor (see Figure S1 in the Supporting Information). After the reaction completion, 1,4-diacetoxybenzene (73 mg, 0.375 mmol) was added, and an aliquot of the mixture was diluted with CDCl_3_ and analyzed using NMR spectroscopy. Then, the reaction mixture was diluted with water (20 mL) and extracted with CH_2_Cl_2_ (3 × 10 mL). Combined organic layers were washed with saturated aqueous NaCl (10 mL), dried over Na_2_SO_4_, and concentrated under reduced pressure. The residue was chromatographed on silica gel using CH_2_Cl_2_–MeOH mixtures (100:1–100:3 *v/v*) as eluents. The products were found to be partially hydrolyzed during purification, which reduced the preparative yield. The NMR spectra of the reaction mixtures are also given in the Supporting Information.

*Diethyl (1,10-phenanthrolin-5-yl)phosphonate (***2***)* [11] was obtained according to the general procedure from 5-bromo-1,10-phenanthroline **1** (96 mg, 0.375 mmol) after 38 h of irradiation. Yield 51% (NMR). ^1^H NMR (CDCl_3_, 400 MHz): δ 9.23 (dd, ^3^*J* = 4.3, ^4^*J* = 1.8 Hz, 1H, 2-H), 9.19 (dd, ^3^*J* = 4.3, ^4^*J* = 1.6 Hz, 1H, 9-H), 8.85 (dd, ^3^*J* = 8.5, ^4^*J* = 1.7 Hz, 1H, 4-H), 8.60 (d, ^3^*J*_H,P_ = 17.4 Hz, 1H, 5-H), 8.31 (dd, ^3^*J* = 8.1, ^4^*J* = 1.8 Hz, 1H, 4-H), 7.68–7.64 (m, 2H, 3,8-H), 4.27–4.04 (m. 4H, OCH_2_), 1.27 (t, ^3^*J* = 7.0, OCH_2_CH_3_). ^31^P{^1^H} NMR (CDCl_3_, 162.5 MHz): δ 16.5 (s, 1P). The obtained spectra are consistent with the literature data.
*Diethyl (1,10-phenanthrolin-4-yl)phosphonate (***7***)* [11] was obtained according to the general procedure from 4-bromo-1,10-phenanthroline **3** (96 mg, 0.375 mmol) after 56 h of irradiation. Rhodamine 6G (27 mg, 15 mol.%) was used instead of Eosin Y. Yield 42% (NMR). ^1^H NMR (CDCl_3_, 400 MHz): δ 9.17 (dd, ^3^*J* = 4.3, ^4^*J*_H,P_ = 4.2 Hz, 1H, 2-H), 9.09 (dd, ^3^*J* = 4.3, ^4^*J* = 1.6 Hz, 1H, 9-H), 8.40 (d, ^3^*J* = 9.2 Hz, 1H, 5-H), 8.16 (dd, ^3^*J* = 8.1, ^4^*J* = 1.6 Hz, 1H, 7-H), 8.06 (dd, ^3^*J*_H,P_ = 15.4 Hz, ^3^*J* = 4.3, 1H, 3-H), 7.82 (d, ^3^*J* = 9.2 Hz, 1H, 6-H), 7.54 (dd, ^3^*J* = 8.1, ^3^*J* = 4.3 Hz, 1H, 7-H), 4.20–4.05 (m. 4H, OCH_2_), 1.22 (t, ^3^*J* = 7.1, OCH_2_CH_3_). ^31^P{^1^H} NMR (CDCl_3_, 162.5 MHz): δ 14.8 (s, 1P). The obtained spectra are consistent with the literature data.
*Diethyl (1,10-phenanthrolin-3-yl)phosphonate (***8***)* [11] was obtained according to the general procedure from 3-bromo-1,10-phenanthroline **4** (96 mg, 0.375 mmol) after 90 h of irradiation. Yield 26% (NMR). ^1^H NMR (CDCl_3_, 400 MHz): δ 9.41 (dd, ^4^*J*_H,P_ = 4.8 Hz, ^4^*J* = 1.7, 1H, 2-H), 9.26 (dd, ^3^*J* = 4.3, ^4^*J* = 1.7 Hz, 1H, 9-H), 8.77 (dd, ^3^*J*_H,P_ = 14.5 Hz, ^4^*J* = 2.0, 1H, 7-H), 8.31 (dd, ^3^*J* = 8.1, ^4^*J* = 1.7 Hz, 1H, 7-H), 7.89 (d, ^3^*J* = 9.4 Hz, 1H, 5-H), 7.86 (d, ^3^*J* = 9.4 Hz, 1H, 6-H), 7.73 (dd, ^3^*J* = 8.1 Hz, ^3^*J* = 4.3, 1H, 8-H), 4.28–4.11 (m. 4H, OCH_2_), 1.34 (t, ^3^*J* = 7.1, OCH_2_CH_3_). ^31^P{^1^H} NMR (CDCl_3_, 162.5 MHz): δ 15.7 (s, 1P). The obtained spectra are consistent with the literature data.
*Diethyl (2-chloro-1,10-phenanthrolin-6-yl)phosphonate (***20***)* was obtained according to the general procedure from 6-bromo-2-chloro-1,10-phenanthroline **14** (110 mg, 0.375 mmol) after 40 h of irradiation. Yield 41% (NMR), preparative yield 26 mg (20%), yellow oil. ^1^H NMR (CDCl_3_, 400 MHz) δ 9.25 (dd, 1H, ^3^*J* = 4.3, ^4^*J* = 1.6, 7-H), 8.88 (dd, 1H, ^3^*J* = 8.4, ^4^*J* = 1.6, 9-H), 8.62 (d, 1H, ^3^*J*_H,P_ = 17.3, 5-H), 8.28 (d, 1H, ^3^*J* = 8.4, 4-H), 7.69 (d, 1H, ^3^*J* = 8.4, 3-H), 7.71 (dd, 1H, ^3^*J* = 8.4, ^3^*J* = 4.3, 8-H), 4.31–4.10 (m, 4H, OCH_2_), 1.33 (t, 6H, ^3^*J* = 7.0, OCH_2_CH_3_). ^13^C{^1^H} NMR (CDCl_3_, 100.6 MHz) δ 153.9 (1C), 151.0 (1C), 145.0 (d, 1C, *J*_C,P_ = 11.6), 139.7 (1C), 135.7 (d, 1C, *J*_C,P_ = 9.2), 135.3 (d, 1C, *J*_C,P_ = 2.4), 127.8 (d, 1C,), 125.3 (d, 1C, ^3^*J*_C,P_ = 18.8), 125.2 (d, 1C, ^1^*J*_C,P_ = 183.9), 125.1 (1C), 123.7 (1C), 62.7 (2C, ^3^*J*_C,P_ = 5.0, OCH_2_), 16.3 (2C, ^3^*J*_C,P_ = 5.0, CH_3_). ^31^P{^1^H} NMR (CDCl_3_, 162.5 MHz) δ 16.3 (s, 1P). IR (neat, ν_max_, cm^−1^): 621, 635, 666, 699, 732, 743, 886, 965, 1014, 1042, 1092, 1137, 1162, 1197, 1231, 1245 (P=O), 1296, 1375, 1389, 1436, 1475, 1497, 1557, 1581, 1603, 1662, 1717, 2867, 2908, 2930, 3044. MALDI-TOF: *m*/*z* found 351.0638. C_16_H_17_ClN_2_O_3_P. Calcd. 351.0660 [M + H]^+^.
*Diethyl (2-methoxy-1,10-phenanthrolin-6-yl)phosphonate (***22***)* was obtained according to the general procedure from 6-bromo-2-methoxy-1,10-phenanthroline **15** (108 mg, 0.375 mmol) after 32 h of irradiation. Yield: 34% (NMR), preparative yield 19 mg (15%), yellow oil. ^1^H NMR (CDCl_3_, 400 MHz) δ 9.26 (dd, ^3^*J* = 4.3, ^4^*J* = 1.6 Hz, 1H, 9-H), 8.94 (dd, ^3^*J* = 8.5, ^4^*J* = 1.6 Hz, 1H, 7-H), 8.60 (d, ^3^*J*_H,P_ = 17.0 Hz, 1H, 5-H), 8.20 (d, ^3^*J* = 8.7 Hz, 1H, 4-H), 7.71 (dd, ^3^*J* = 8.5, ^3^*J* = 4.2 Hz, 1H, 8-H), 7.19 (d, ^3^*J* = 8.7 Hz, 1H, 3-H), 4.36 (s, 3H, OMe), 4.30–4.07 (m. 4H, OCH_2_), 1.31 (t, ^3^*J* = 7.0, OCH_2_CH_3_). ^13^C{^1^H} NMR (CDCl_3_, 100.6 MHz) δ 165.0 (1C), 149.7 (1C), 146.2 (1C), 139.8 (1C), 136.7 (d, 1C, *J*_C,P_ = 9.2), 136.1 (1C), 123.1 (1C), 122.9 (1C), 122.9 (1C), 120.6 (d, ^1^*J*_C,P_ = 186.1), 115.0 (1C), 62.5 (2C, ^3^*J*_C,P_ = 5.2, OCH_2_), 55.5 (1C, OMe), 16.4 (2C, ^3^*J*_C,P_ = 6.5, CH_3_). ^31^P{^1^H} NMR (CDCl_3_, 162.5 MHz) δ 17.5 (s, 1P). IR (neat, ν_max_, cm^−1^) 607, 627, 662, 669, 754, 805, 825, 980 (P-O), 1018(P–O–C), 1048 (C–O–C), 1087 (C–O–C), 1145 (C–O–C), 1163, 1200, 1235, 1249 (P–O–C), 1274 (P=O), 1357 (C–N), 1393, 1425, 1469, 1488, 1504, 1560 (C=N), 1592 (C=N), 1610 (C=N), 2930, 2980. ESI-TOF: *m*/*z* found 347.1159. C_17_H_20_N_2_O_4_P. Calcd. 347.1155 [M + H]^+^.
*Diethyl (4-methoxy-1,10-phenanthrolin-6-yl)phosphonate (***23***)* was obtained according to the general procedure from 6-bromo-4-methoxy-1,10-phenanthroline **19** (108 mg, 0.375 mmol) after 16 h of irradiation. Yield 40% (NMR), preparative yield 28 mg (21%), yellow oil. ^1^H NMR (CDCl_3_, 400 MHz) δ 9.22 (dd, 1H, ^3^*J* = 4.2, ^4^*J* = 1.5, 7-H), 9.12 (d, ^3^*J* = 5.3, 1H, 2-H), 9.06 (d, 1H, ^3^*J*_H,P_ = 17.5, 5-H), 8.91 (dd, 1H, ^3^*J* = 8.4, ^4^*J* = 1.5, 9-H), 7.70 (dd, 1H, ^3^*J* = 8.4, ^3^*J* = 4.2, 8-H), 7.08 (d, 1H, ^3^*J* = 5.3, 3-H), 4.31–4.08 (m, 4H, OCH_2_), 4.13 (s, 3H, OMe), 1.33 (t, 6H, ^3^*J* = 7.0, OCH_2_CH_3_). ^13^C{^1^H} NMR (CDCl_3_, 100.6 MHz) δ 163.1 (1C), 153.7 (1C), 150.5 (1C), 145.9 (d, 1C, *J*_C,P_ = 12.0), 135.2 (d, 1C, *J*_C,P_ = 2.8), 130.7 (d, 1C, *J*_C,P_ = 10.4), 127.5 (1C, *J*_C,P_ = 10.7), 123.6 (1C), 123.3 (1C), 120.9 (d, ^1^*J*_C,P_ = 168.1), 119.0 (d, *J*_C,P_ = 19.4), 103.7 (1C), 62.6 (2C, ^3^*J*_C,P_ = 4.8, OCH_2_), 56.1 (1C, OMe), 16.3 (2C, ^3^*J*_C,P_ = 6.2, CH_3_). ^31^P{^1^H} NMR (CDCl_3_, 162.5 MHz): δ 17.5 (s, 1P). IR (neat, ν_max_, cm^−1^) 621, 632, 698, 733, 754, 773, 803, 834, 854, 892, 962, 1012, 1044, 1086, 1108, 1157, 1198, 1213, 1248 (P=O), 1270, 1286, 1303, 1348, 1370, 1399, 1413, 1437, 1455, 1487, 1509, 1564, 1586, 1727, 2588, 2927, 2979. MALDI-TOF: *m*/*z* found 347.1192. C_17_H_20_N_2_O_4_P. Calcd. 347.1155 [M + H]^+^.

## 4. Conclusions

To sum up, we have studied the photoredox-catalyzed phosphonylation of bromo-substituted 1,10-phenanthrolines with triethyl phosphite under visible light irradiation. We have revealed that the reactivity of bromo-substituted phenanthrolines strongly depends on the position of the bromine atom in the heterocyclic system. The nature and position of additional substituents in 1,10-phenanthroline core also have a strong effect on the reactivity of the substrate. In the case of 1,10-phenanthrolines containing bromine at position 5, medium yields were achieved in the presence of the Eosin Y photocatalyst and DIPEA as a sacrificial electron donor under irradiation with blue light (450 nm). The reaction proceeds under mild conditions without the application of transition metals and affords target products in 26–51% yields. It has been shown that despite the modest yields and long reaction time, this approach is an acceptable alternative to Pd-catalyzed phosphonylation in the case of the presence of electron-donor substituents in the phenanthroline core.

## Figures and Tables

**Table 1:** Photoredox-catalyzed phosphonylation of 5-bromo-1,10-phenanthroline.

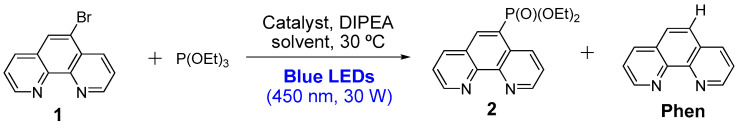						
Entry ^1^	Catalysts (mol%)	Solvent	Time (h)	Conversion (%) ^2^	Yield of 2 (%) ^2^	Yield of Phen (%) ^2^
1 ^3^	Rhodamine 6G (10)	DMSO	16	0	n/d	n/d
2 ^4^	Rhodamine 6G (10)	DMSO	16	0	n/d	n/d
3	Rhodamine 6G (10)	DMSO	16	17	13	4
4	4-CzIPN (10)	DMSO	22	50	27	10
5	4-CzIPN (10+5) ^5^	DMSO	22+16 ^5^	64	34	14
6	Ru(bpy)_3_Cl_2_ (2)	DMSO	16	68	39	12
7	Eosin Y (10)	DMSO	16	83	47	14
8	Eosin Y (10+5) ^5^	DMSO	16+22 ^5^	96	52	16
9	Fluorescein (10)	DMSO	16	66	40	9
10	Fluorescein (10+5) ^5^	DMSO	16+16 ^5^	93	46	9
11	Eosin Y (15)	DMSO	22	94	51	14
12	Eosin Y (15)	DMF	22	96	26	28
13	Eosin Y (15)	MeCN	22	47	6	8

^1^ Reaction conditions: 5-Bromo-1,10-phenanthroline (0.375 mmol), catalyst, solvent (2 mL), P(OEt)_3_ (1.125 mmol, 195 μL, 3 equiv.), DIPEA (0.825 mmol, 147 μL, 2.2 equiv.), 30 W blue LEDs (450 nm), 30 °C, argon atmosphere. ^2^ The yield and conversion were determined with NMR ^1^H analysis of reaction mixtures, 1,4-diacetoxybenzene was used as an internal standard. ^3^ The reaction was performed with 12 W green LEDs (525 nm) at r.t. ^4^ The reaction was performed with 12 W blue LEDs (455 nm) at r.t. ^5^ Additional portion of the photocatalyst (5 mol%) was added to the reaction mixture, and irradiation was continued for additional time.

## Data Availability

The data presented in this work are available in this article and the Supplementary Materials.

## Supplementary Materials

The following supporting information can be downloaded at: https://www.mdpi.com/article/10.3390/molecules29235558/s1, Figure S1: Photoreactor setup; Table S1: Control experiments; Figure S2: ESI-MS spectrum of the reaction mixture in the presence of 1.5 equiv. of TEMPO after 8 h of irradiation; Table S2: TON values for studied substrates; Figures S3–S14: ^1^H and ^31^P NMR spectra of the reaction mixtures; Figures S15–S36: ^1^H, ^13^C and ^31^P NMR spectra of the new compounds.
