# Supplementary material for: Visible Light Photoredox Catalysis in the Synthesis of Phosphonate-Substituted 1,10-Phenanthrolines
Source: Molecules. 2024 Nov 25;29(23):5558. doi: 10.3390/molecules29235558 (PMC11643584; doi:10.3390/molecules29235558)
Supplement: Supplementary file 1 [file molecules-29-05558-s001.zip › molecules-3263134-supplementary.pdf]

Supporting information

for the article

Visible light photoredox catalysis in the synthesis of phosphonate-substituted 1,10-phenanthrolines

by
Gleb V. Morozkov, Artem A. Troickiy, Alexei D. Averin,
Alexander Yu. Mitrofanov, Anton S. Abel and Irina P. Beletskaya

Table of content

1. Photoreactor setup S2
2. Control experiments S2
3. NMR spectra of the reaction mixtures S5
4. NMR spectra of the new compounds S11

1. Photoreactor setup

***Hepatochem* ® *PhotoRedOx Box* with 30 W LED** was purchased commercially from HepatoChem co. (https://www.hepatochem.com/), λ_{max}= 450 nm, 30W (Figure S1). The temperature of the reaction mixture after 24 hours of irradiation was measured to be 30 °C.

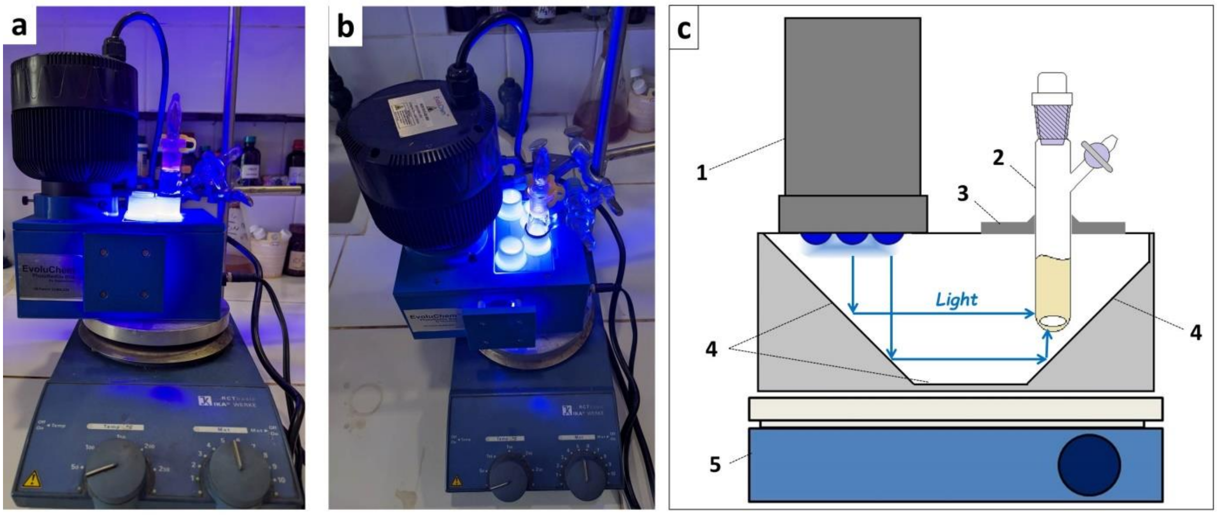

Fig. S1. Photoreactor setup (PhotoRedOx Box): **a, b** – front view of the photoreactor; **c** – schematic representation of photoreactor setup; **1** – Light source (Hepatochem® 30 W Blue LED); **2** – sealed Schlenk tube (diameter 18 mm, the height of the reaction mixture level in the vessel is 20 mm); **3** – plastic tube holder; **4** – reflectors; **5** – magnetic stirrer (IKA® RCT basic).

2. Control experiments

Table S1. Control experiments.

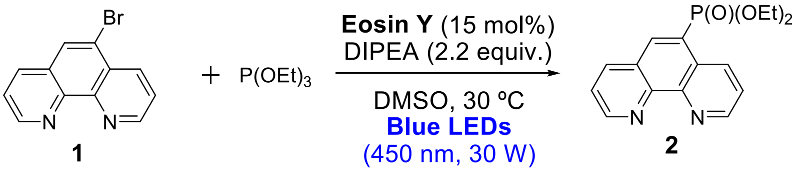

Entry [1]	Deviation from Standard Conditions	Conversion (%) [2]	Yield of 2 (%) [2]
1	none	94	51
2	no irradiation	0	n/d
3	no DIPEA	0	n/d
4	12 W LEDs	0	n/d
5	no photocatalyst, 12 W LEDs	0	n/d
6 [3]	no photocatalyst	36	12
7 [4]	no photocatalyst	34	8 (product **7**)
8	no photocatalyst	38	n/d (product **8**)
9	1.5 equiv. of TEMPO was added	57	19
10	4 equiv. of TEMPO was added	5	n/d

[1] Reaction conditions: 5-Bromo-1,10-phenanthroline **1** (0.375 mmol), Eosin Y (15 mol%), DMSO (2 mL), $P(OEt)_3$ (1.125 mmol, 195 µL, 3 equiv.), DIPEA (0.825 mmol, 147 µL, 2.2 equiv.), blue LEDs, 30 °C, argon atmosphere. [2] The yield and conversion were determined by NMR ^{1}H analysis of reaction mixtures, 1,4-diacetoxybenzene was used as an internal standard. [3] 4-Bromo-1,10-phenanthroline (0.375 mmol) **3** was used instead of **1**. [4] 3-Bromo-1,10-phenanthroline (0.375 mmol) **4** was used instead of **1**.

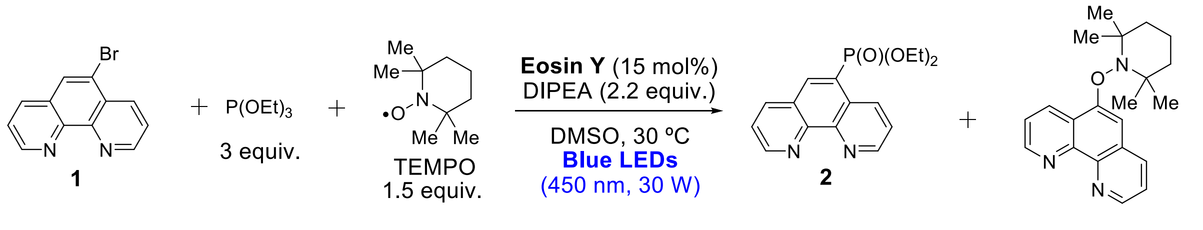

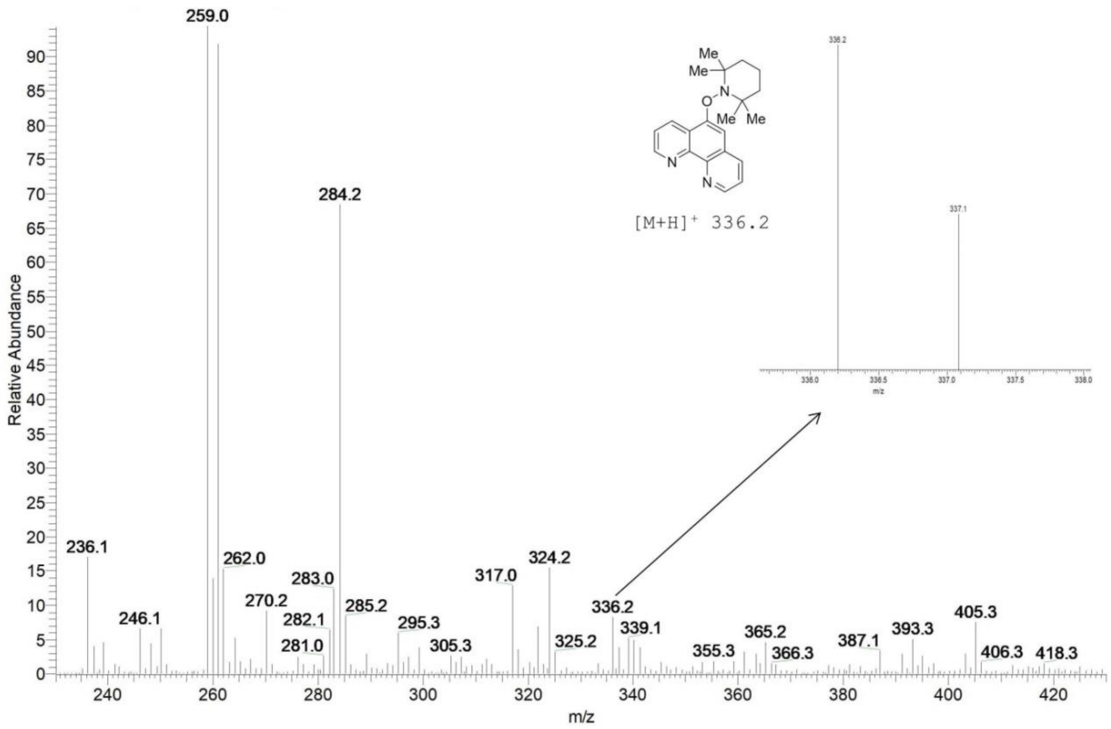

Fig. S2. ESI-MS spectrum of the reaction mixture in the presence of 4 equiv. of TEMPO (Table S1, entry 8) after 8 h of irradiation. The analysis was performed using TSQ Endura spectrometer. Thermo Fisher Scientific, USA). The diluted acetonitrile solution of the sample was introduced through syringe pump directly into the ion source at 10 μL/min. Positive mode, ion transfer tube temperature 275°C, vaporizer temperature 40°C, spray voltage 3.4 kV.

Table S2. TON values for studied substrates.

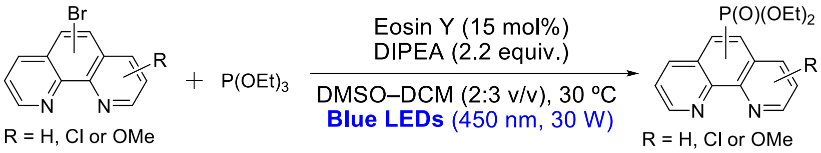

Entry [1]	Starting compound	Product	Yield (%) [2]	TON
1	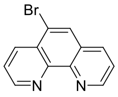 **1**	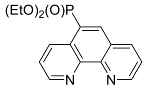 **2**	51	4
2 [3]	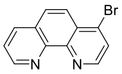 **3**	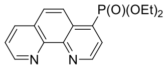 **7**	42	3
3	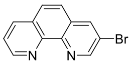 **4**	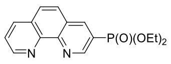 **8**	26	2
4 [4]	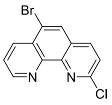 **14**	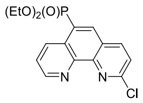 **20**	41	3
5 [4]	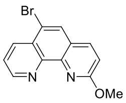 **15**	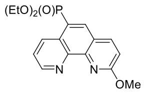 **22**	34	2
6 [4]	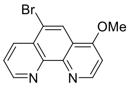 **19**	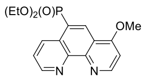 **23**	40	3

Reaction conditions: [1] Bromo-1,10-phenanthroline (0.375 mmol), Eosin Y (15 mol%), DMSO (2 mL), $P(OEt)_3$ (1.125 mmol, 195 μL, 3 equiv.), DIPEA (0.825 mmol, 147 μL, 2.2 equiv.), blue LEDs, 30 °C, argon atmosphere. [2] The yield and conversion were determined by NMR ^{1}H analysis of reaction mixtures, 1,4-diacetoxybenzene was used as an internal standard. [3] Rhodamine 6G (15 mol%) was used as a catalyst instead of Eosin Y. [4] CH_2Cl_2 (3 mL) was added to the mixture.

3. NMR spectra of the reaction mixtures

NMR spectra of the reaction mixture obtained for the compound ***2***

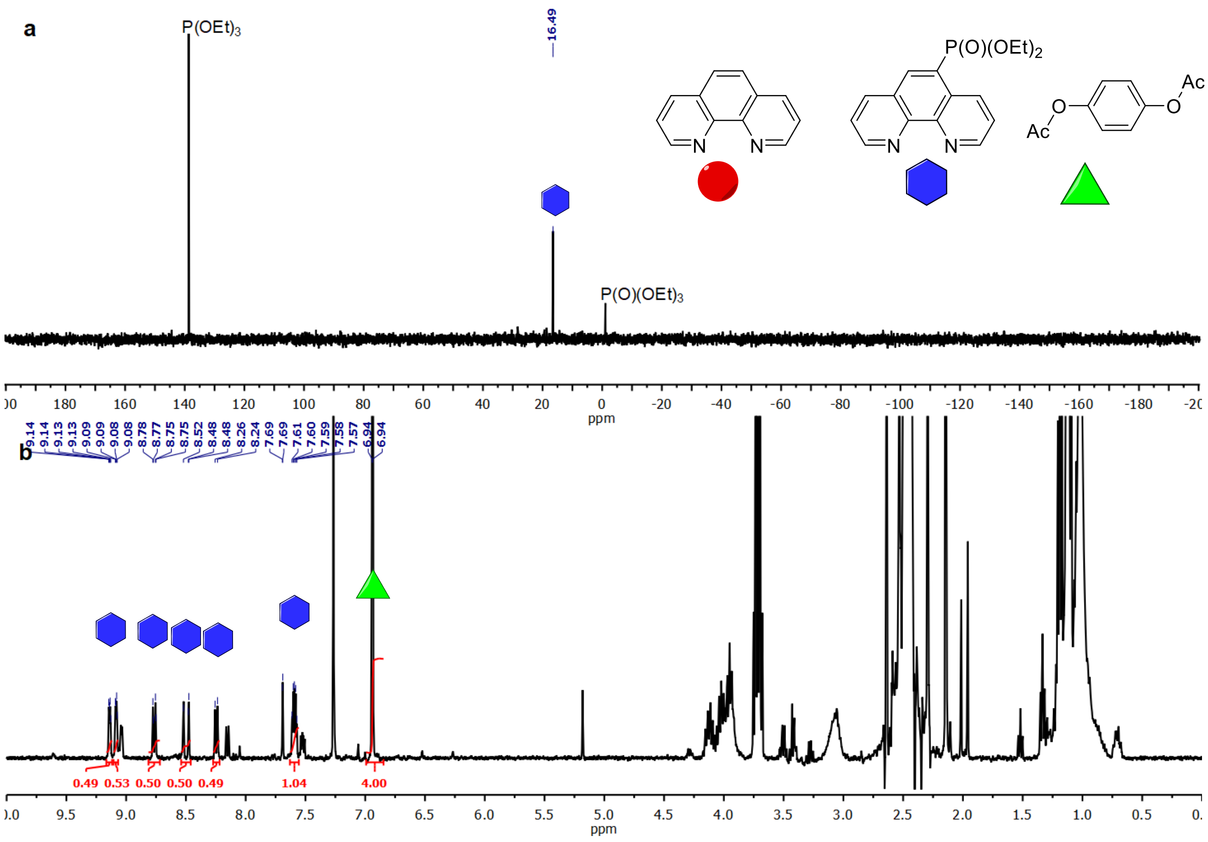

Fig. S3. ${}^{31}P$ (a) and ${}^{1}H$ (b) NMR spectra of the reaction mixture obtained for the compound **2** ($CDCl_3$, 298 K).

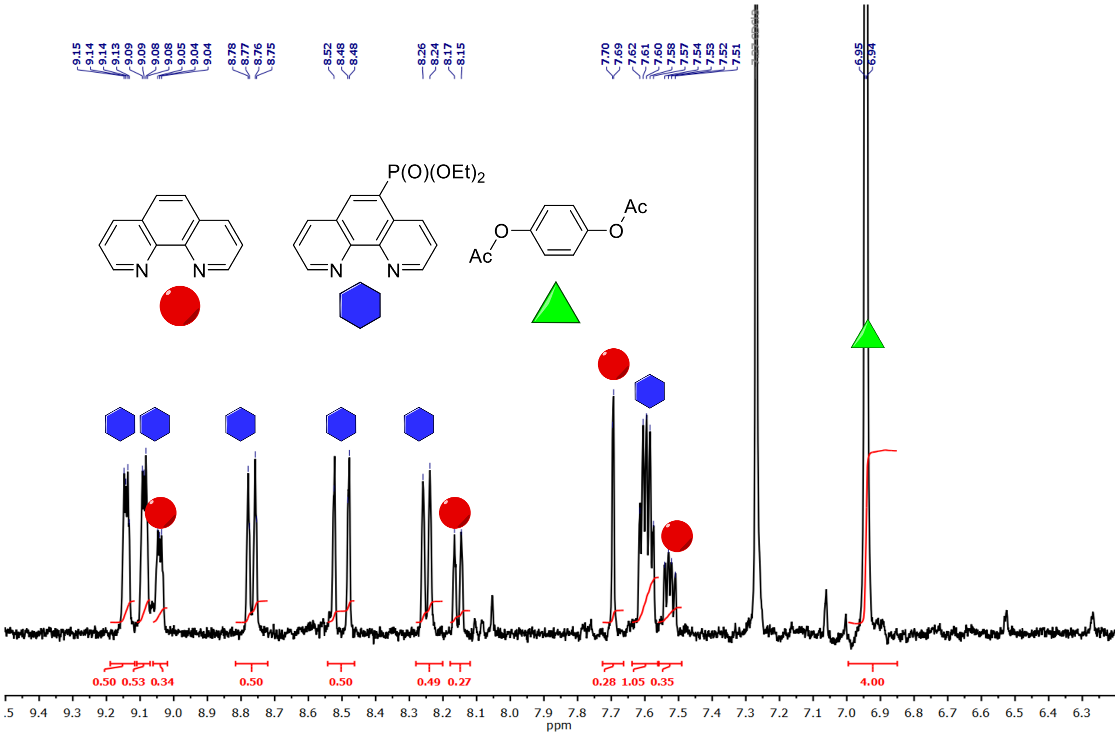

Fig. S4. Aromatic region of ${}^{1}H$ NMR spectra (400 MHz, $CDCl_3$, 298 K) of the reaction mixture obtained for the compound **2**.

*NMR spectra of the reaction mixture obtained for the compound **7***

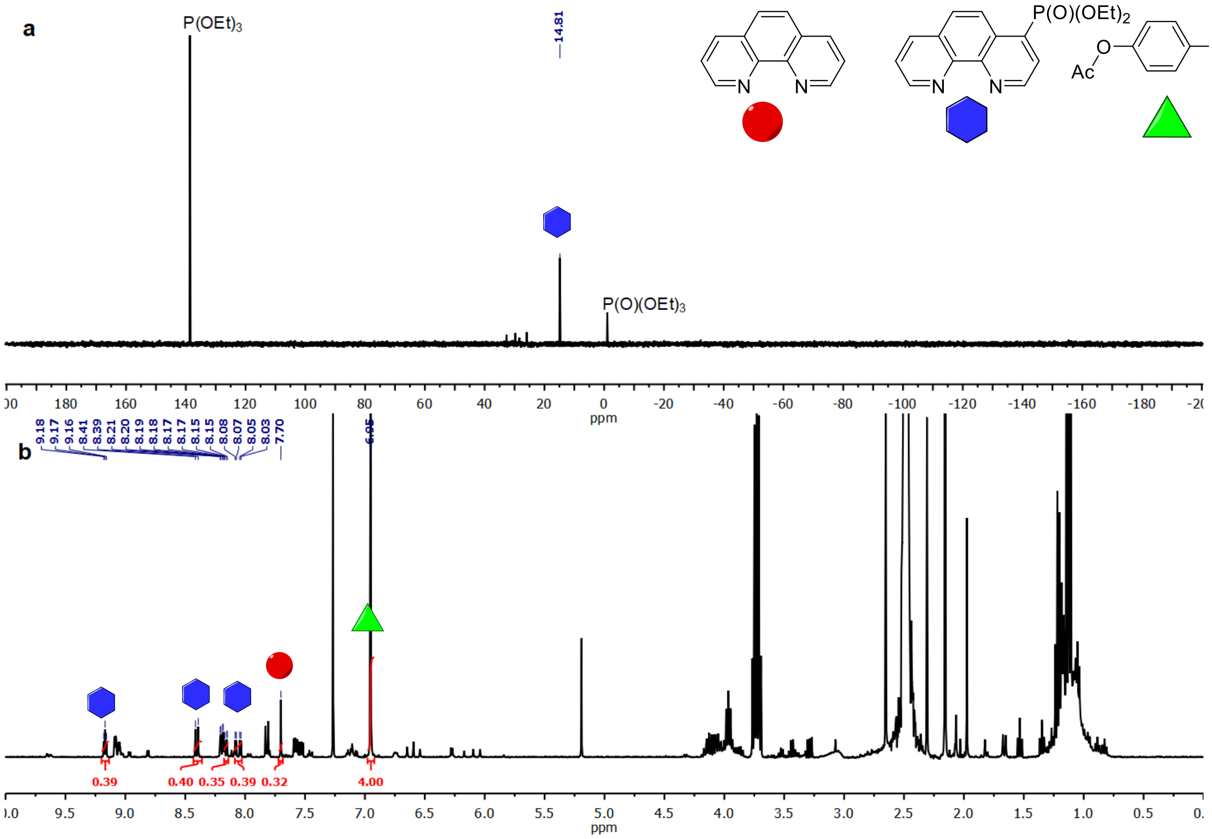

Fig. S5. ^{31}P (a) and ^{1}H (b) NMR spectra of the reaction mixture obtained for the compound **7** (DMSO-D6, 298 K).

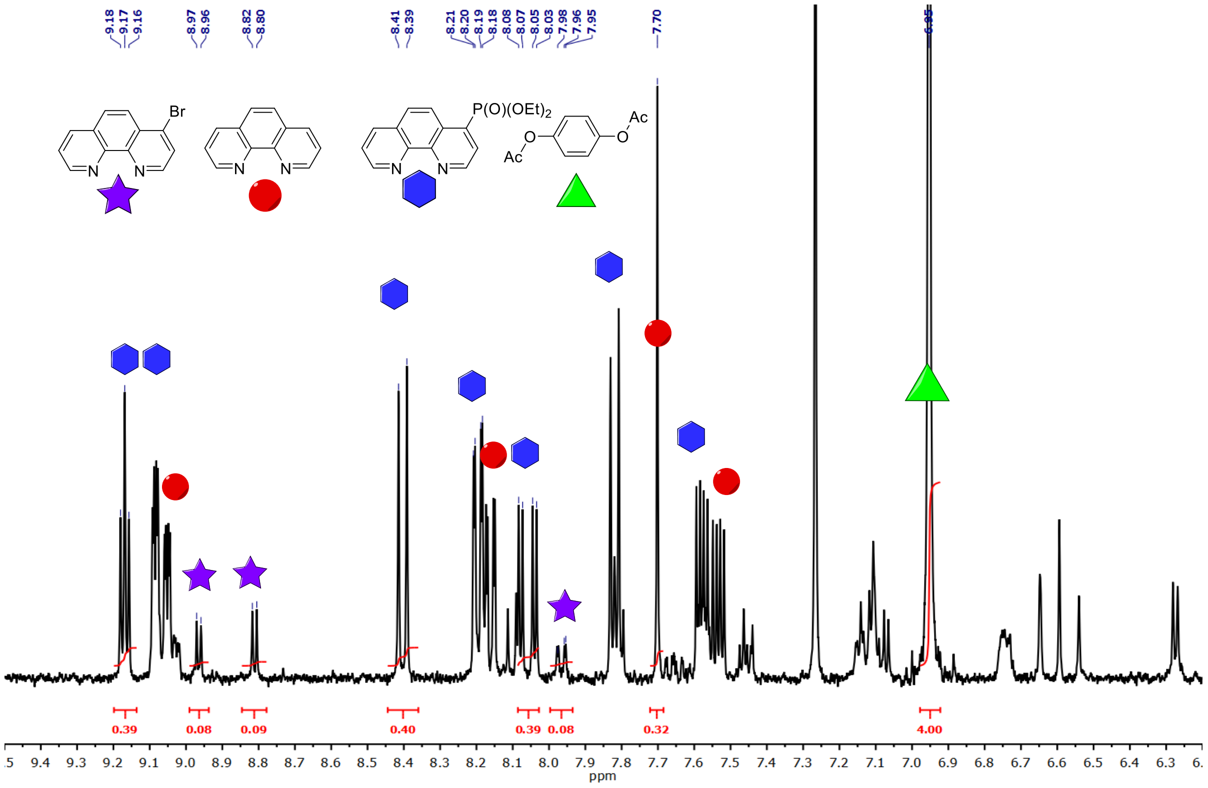

Fig. S6. Aromatic region of ^{1}H NMR spectra (400 MHz, DMSO-D6, 298 K) of the reaction mixture obtained for the compound **7**.

*NMR spectra of the reaction mixture obtained for the compound **8***

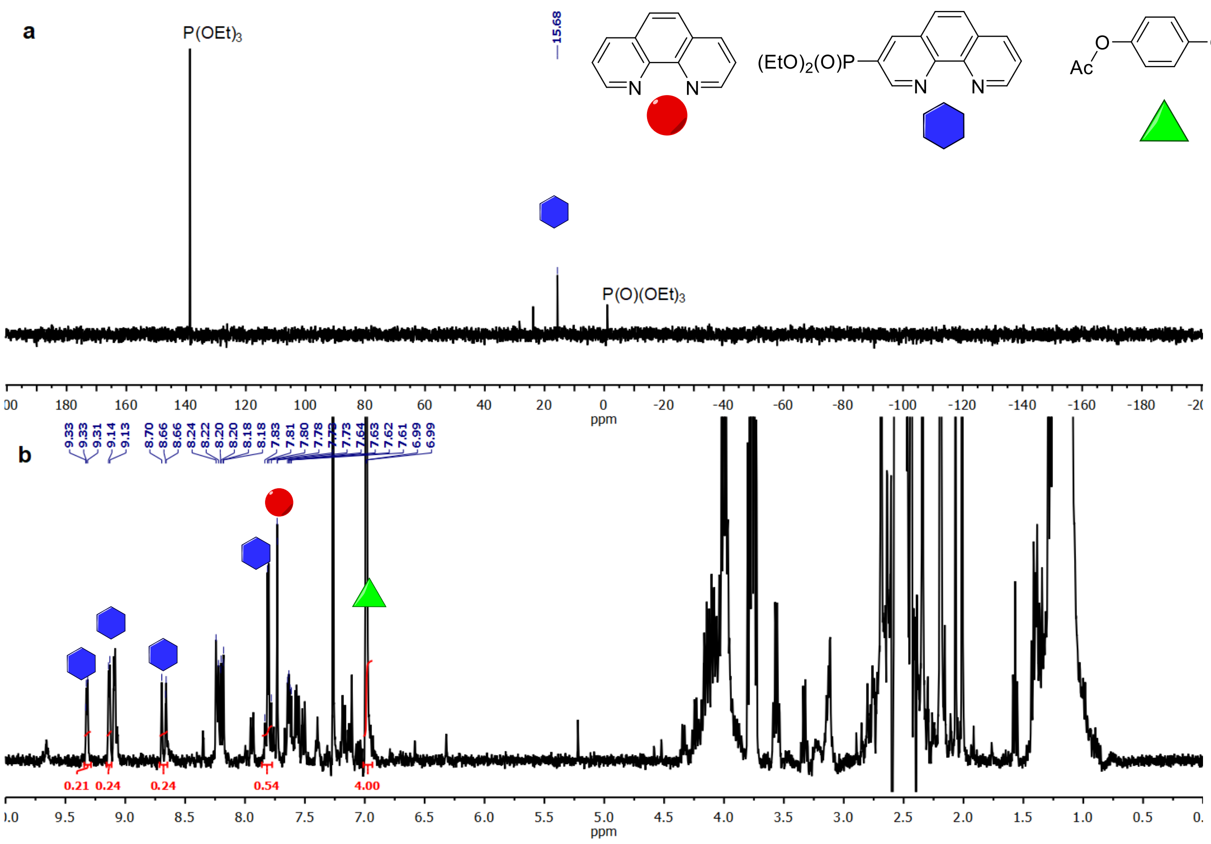

Fig. S7. ^{31}P (a) and ^{1}H (b) NMR spectra ($CDCl_3$, 298 K) of the reaction mixture obtained for the compound **8**.

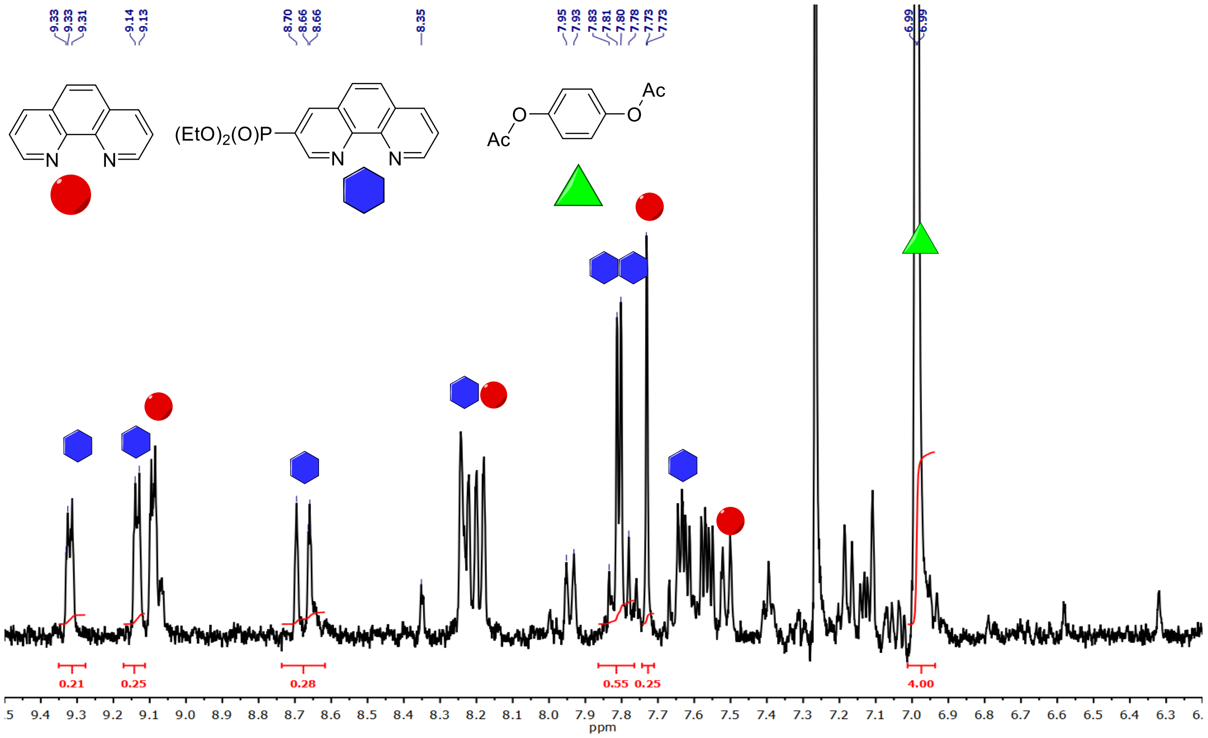

Fig. S8. Aromatic region of ^{1}H NMR spectra (400 MHz, $CDCl_3$, 298 K) of the reaction mixture obtained for the compound **8**.

NMR spectra of the reaction mixture obtained for the compound ***20***

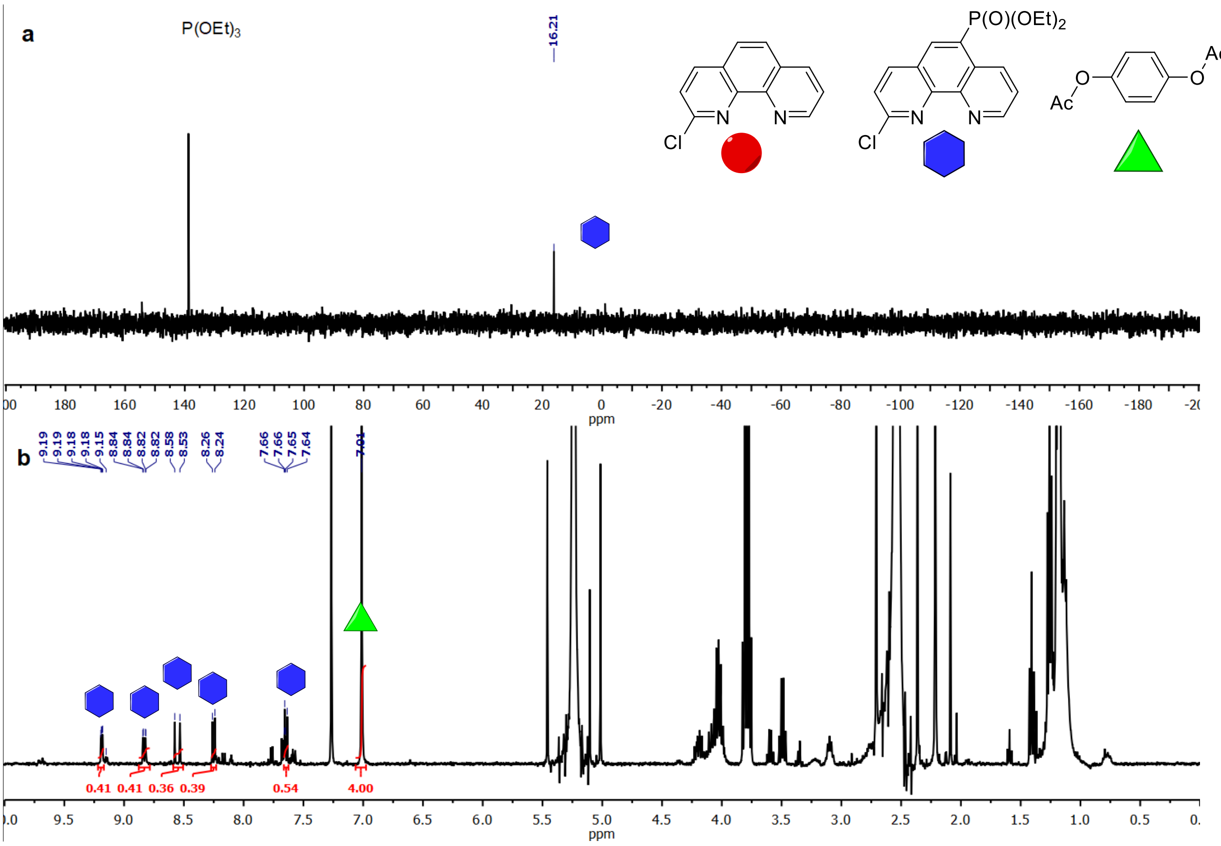

Fig. S9. ^{31}P (a) and ^{1}H (b) NMR spectra ($CDCl_3$, 298 K) of the reaction mixture obtained for the compound **20**.

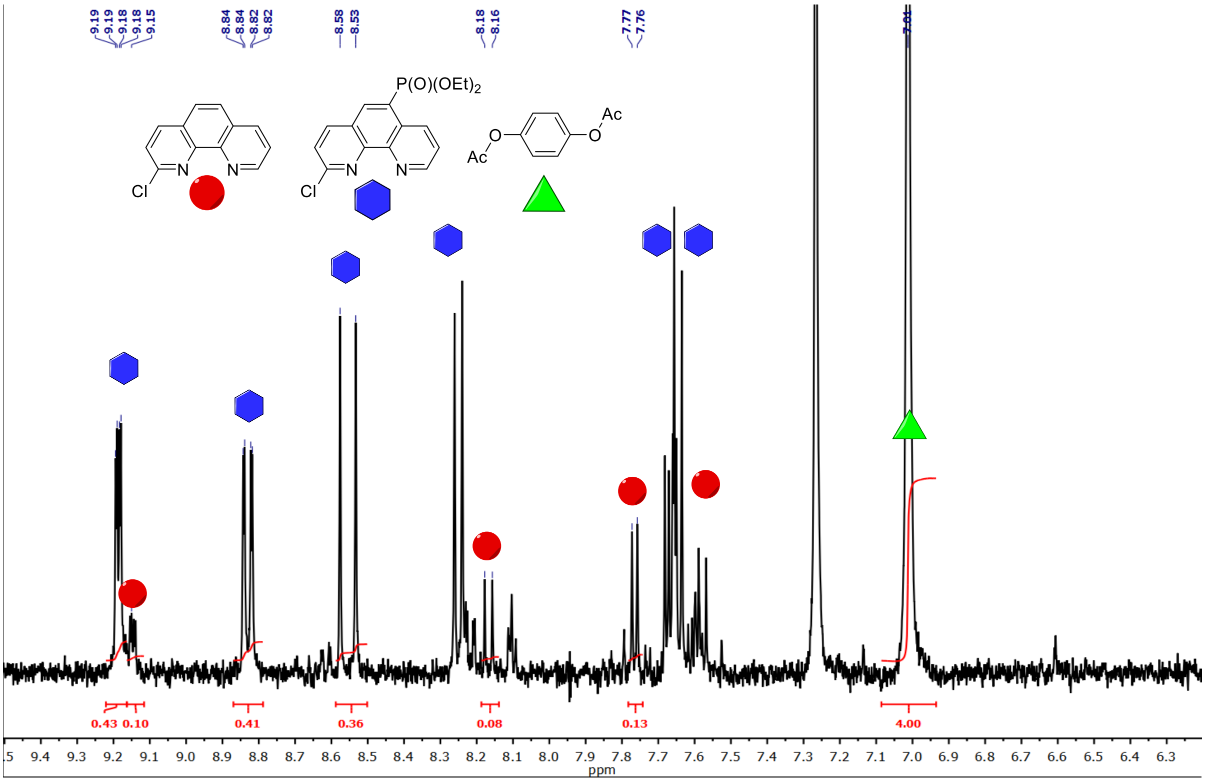

Fig. S10. Aromatic region of ^{1}H NMR spectra (400 MHz, $CDCl_3$, 298 K) of the reaction mixture obtained for the compound **20**.

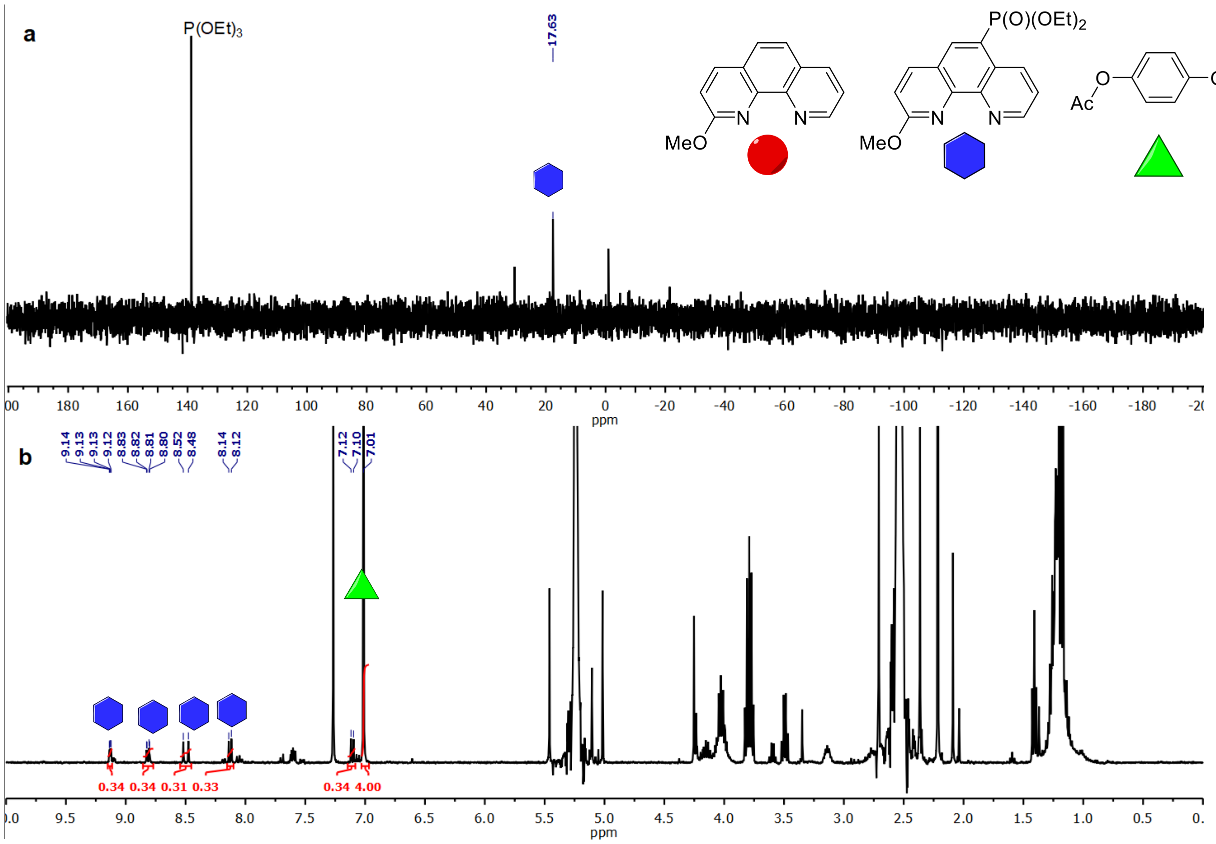

Fig. S11. ^{31}P (a) and ^{1}H (b) NMR spectra ($CDCl_3$, 298 K) of the reaction mixture obtained for the compound **22**.

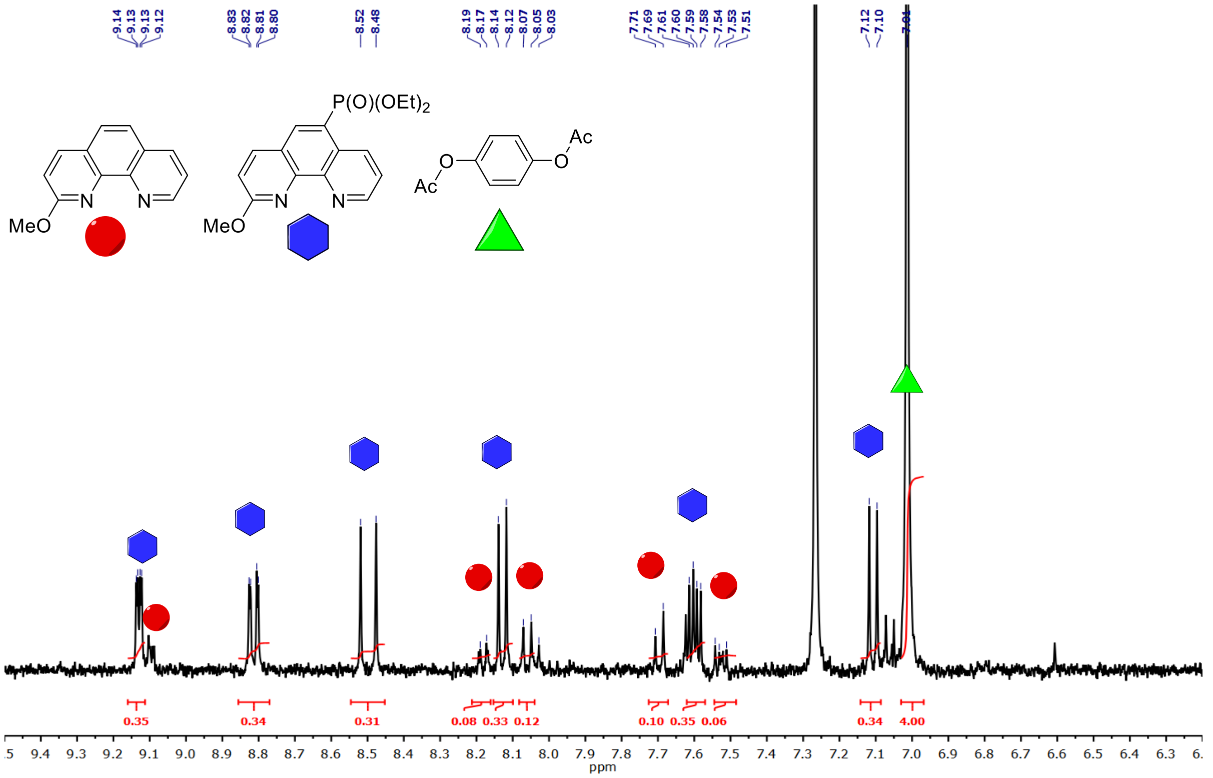

Fig. S12. Aromatic region of ^{1}H NMR spectra (400 MHz, $CDCl_3$, 298 K) of the reaction mixture obtained for the compound **22**.

NMR spectra of the reaction mixture obtained for the compound ***23***

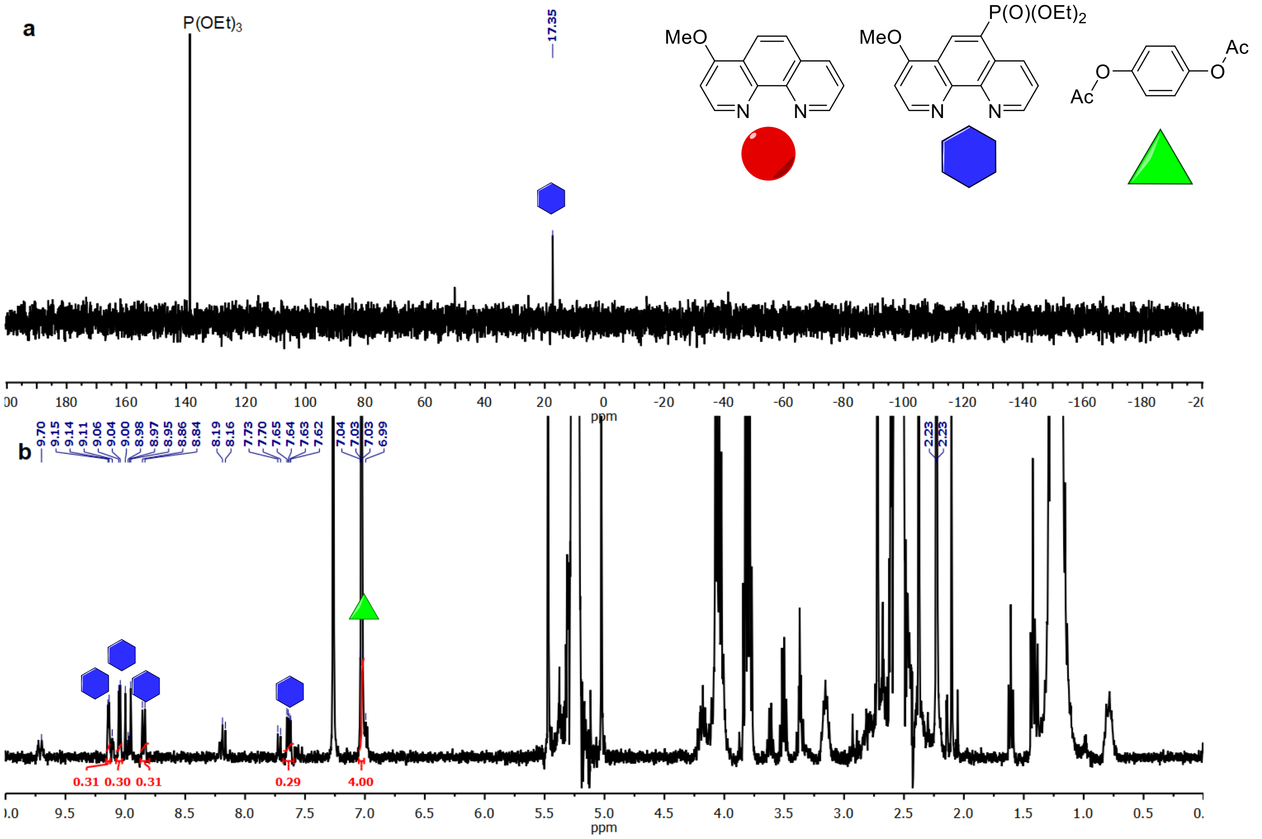

Fig. S13. ^{31}P (a) and ^{1}H (b) NMR spectra ($CDCl_3$, 298 K) of the reaction mixture obtained for the compound **23**.

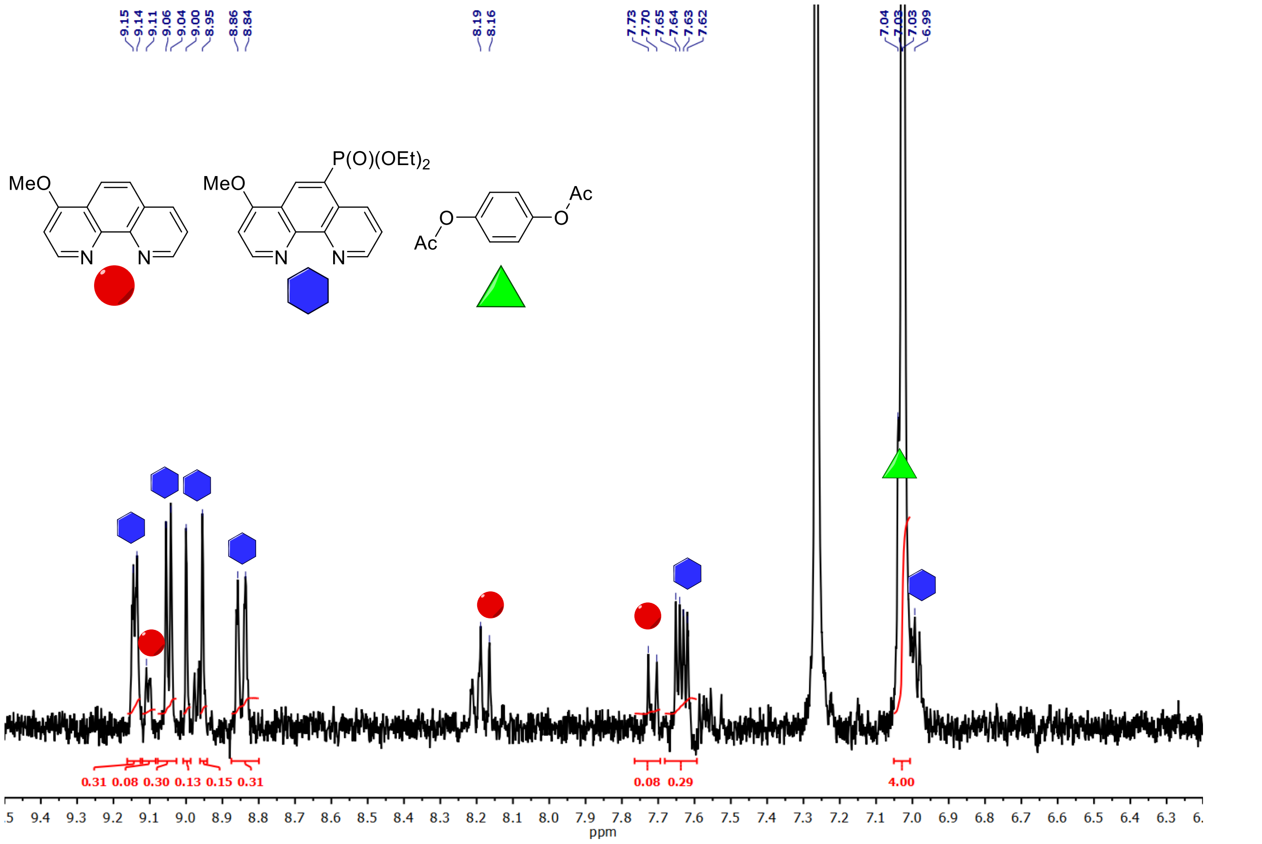

Fig. S14. Aromatic region of ^{1}H NMR spectra (400 MHz, $CDCl_3$, 298 K) of the reaction mixture obtained for the compound **23**.

4. NMR spectra of the new compounds

Spectra of the starting compounds

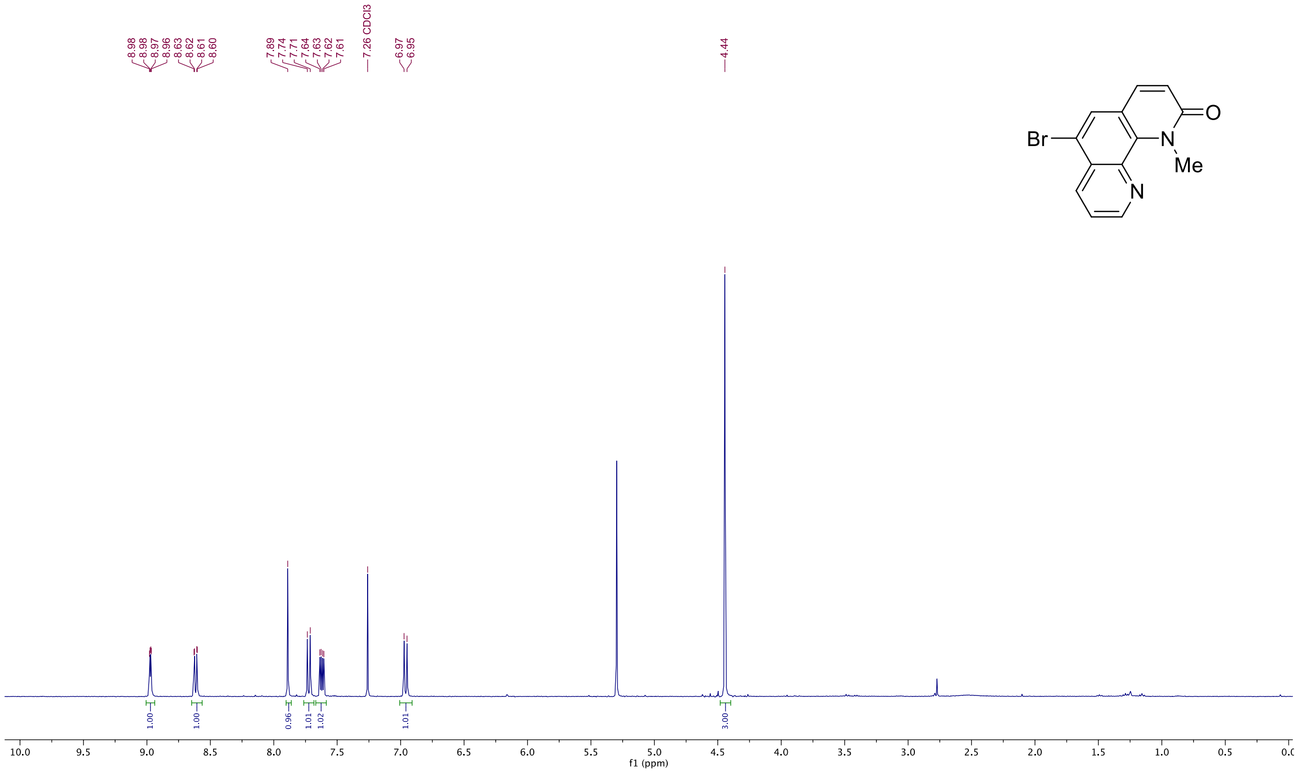

Fig. S15. ^{1}H NMR spectrum of the compound **13** ($CDCl_3$, 400 MHz, 298 K).

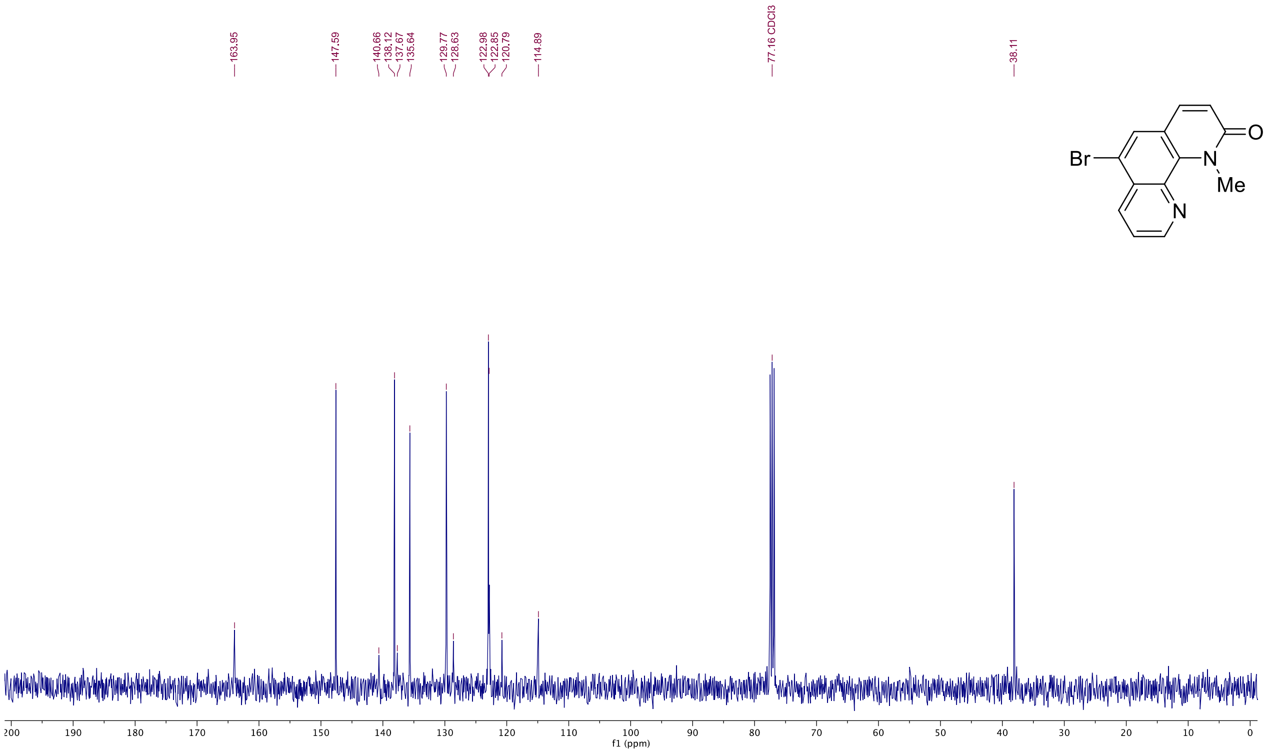

Fig. S16. ^{13}C NMR spectrum of the compound **13** ($CDCl_3$, 100.6 MHz, 298 K).

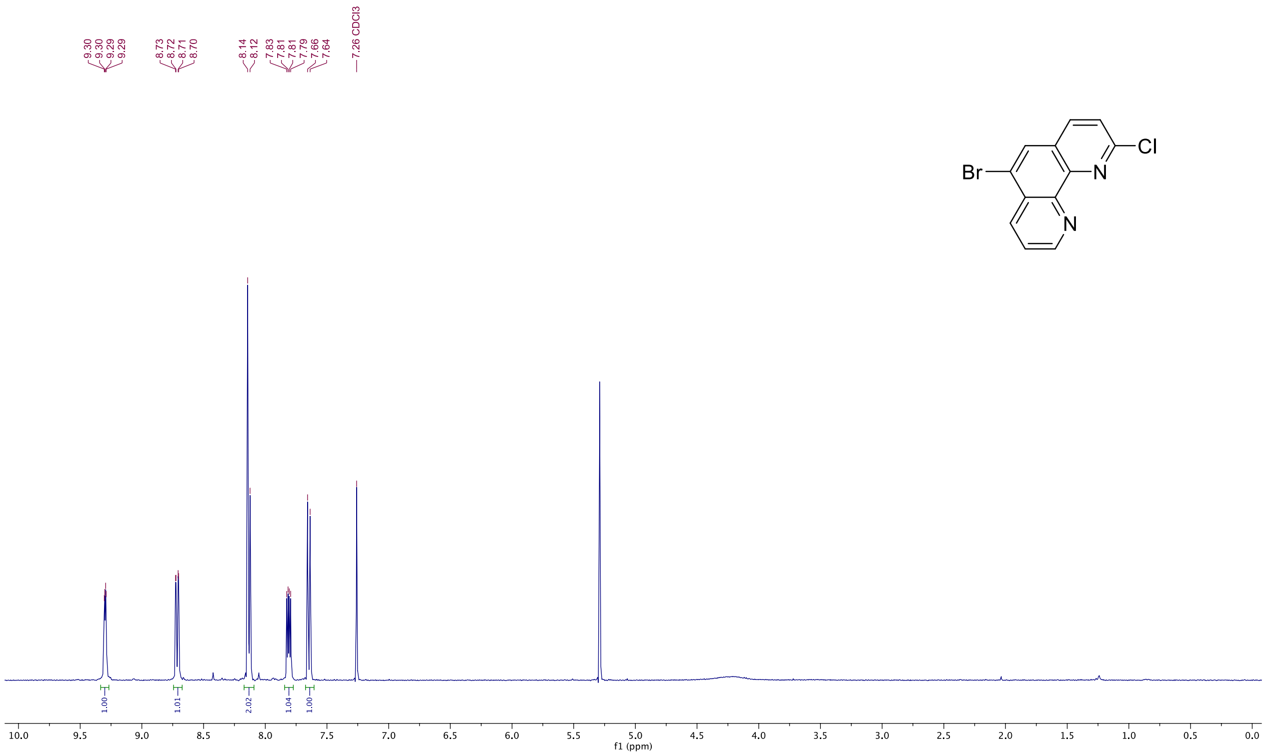

Fig. S17. ^{1}H NMR spectrum of the compound **14** ($CDCl_3$, 400 MHz, 298 K).

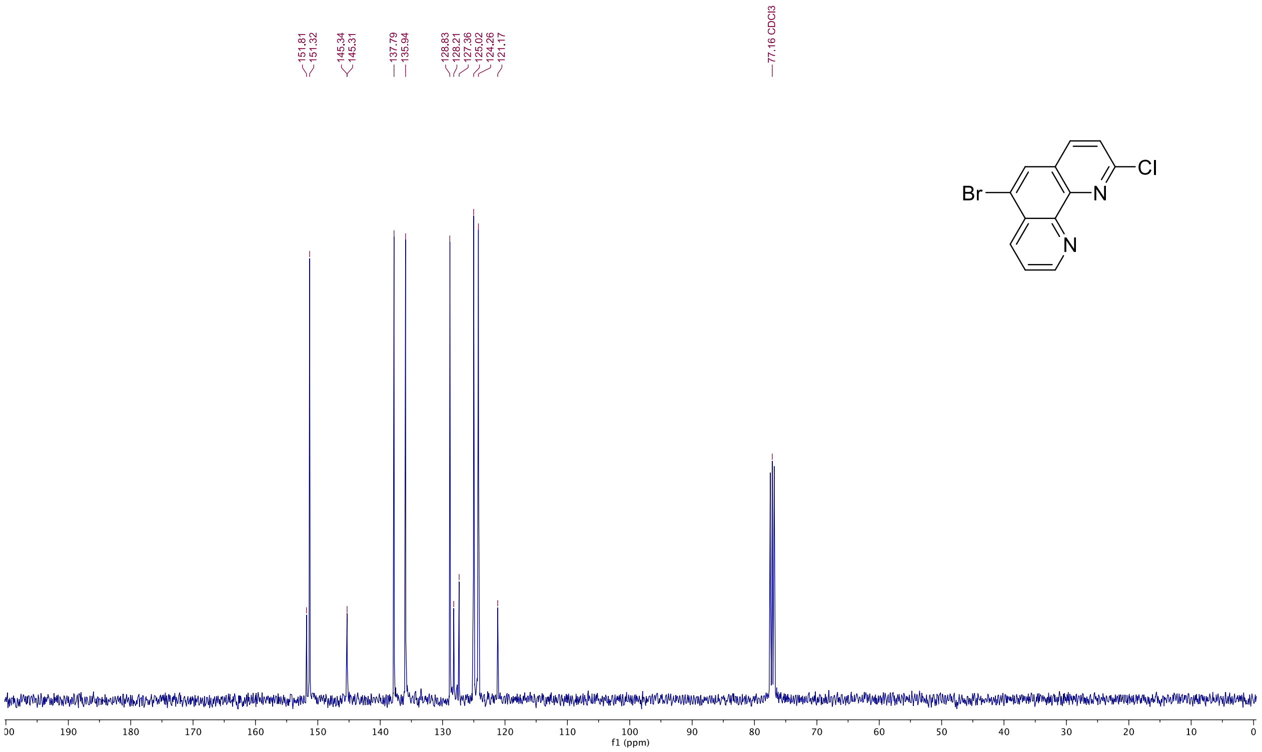

Fig. S18. ^{13}C NMR spectrum of the compound **14** ($CDCl_3$, 100.6 MHz, 298 K).

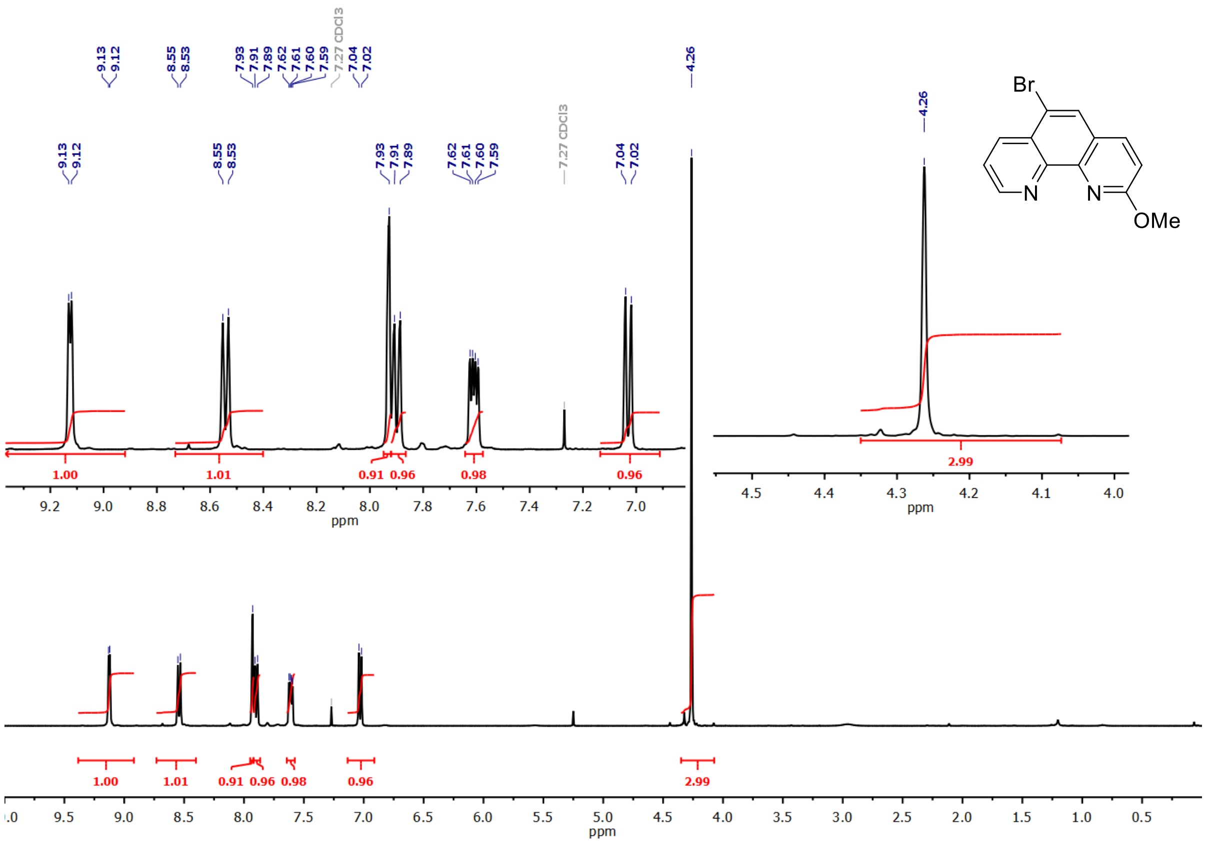

Fig. S19. ^{1}H NMR spectrum of the compound **15** ($CDCl_3$, 400 MHz, 298 K).

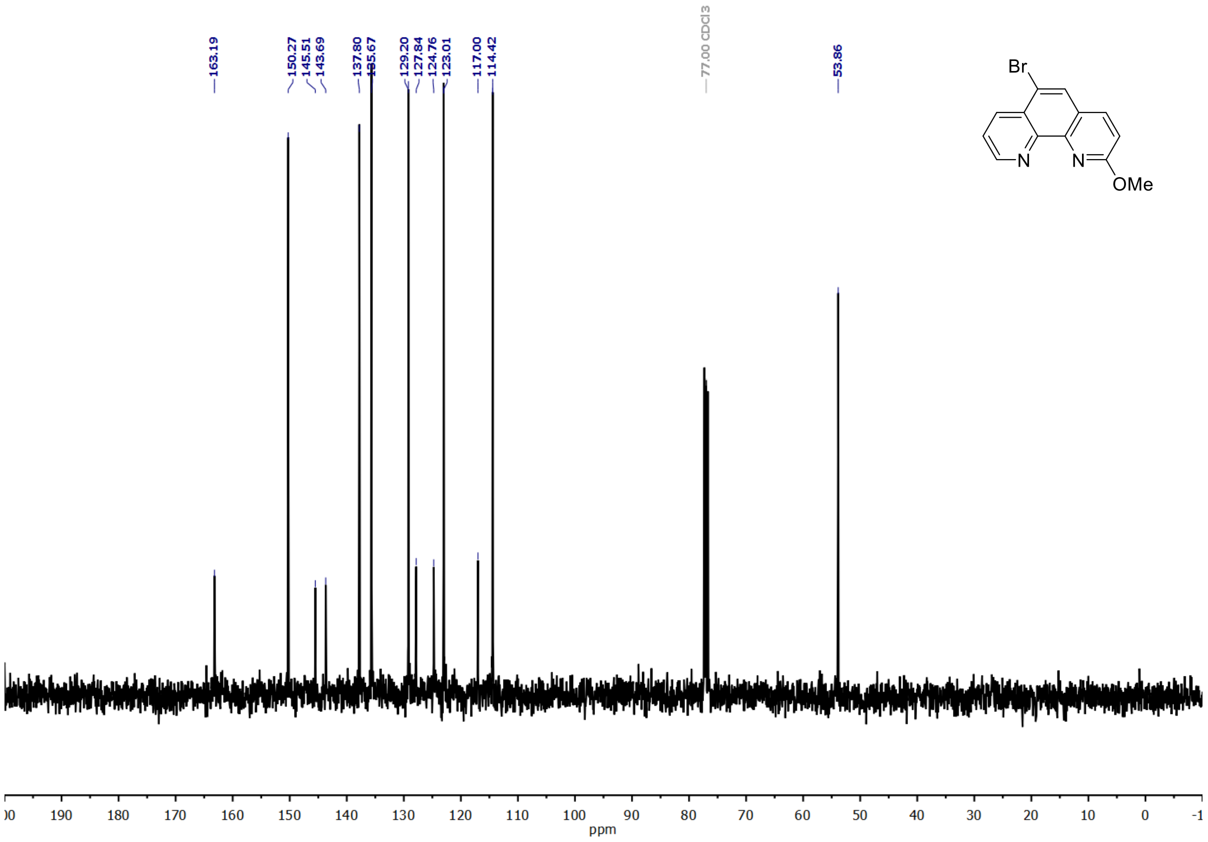

Fig. S20. ^{13}C NMR spectrum of the compound **15** ($CDCl_3$, 100.6 MHz, 298 K).

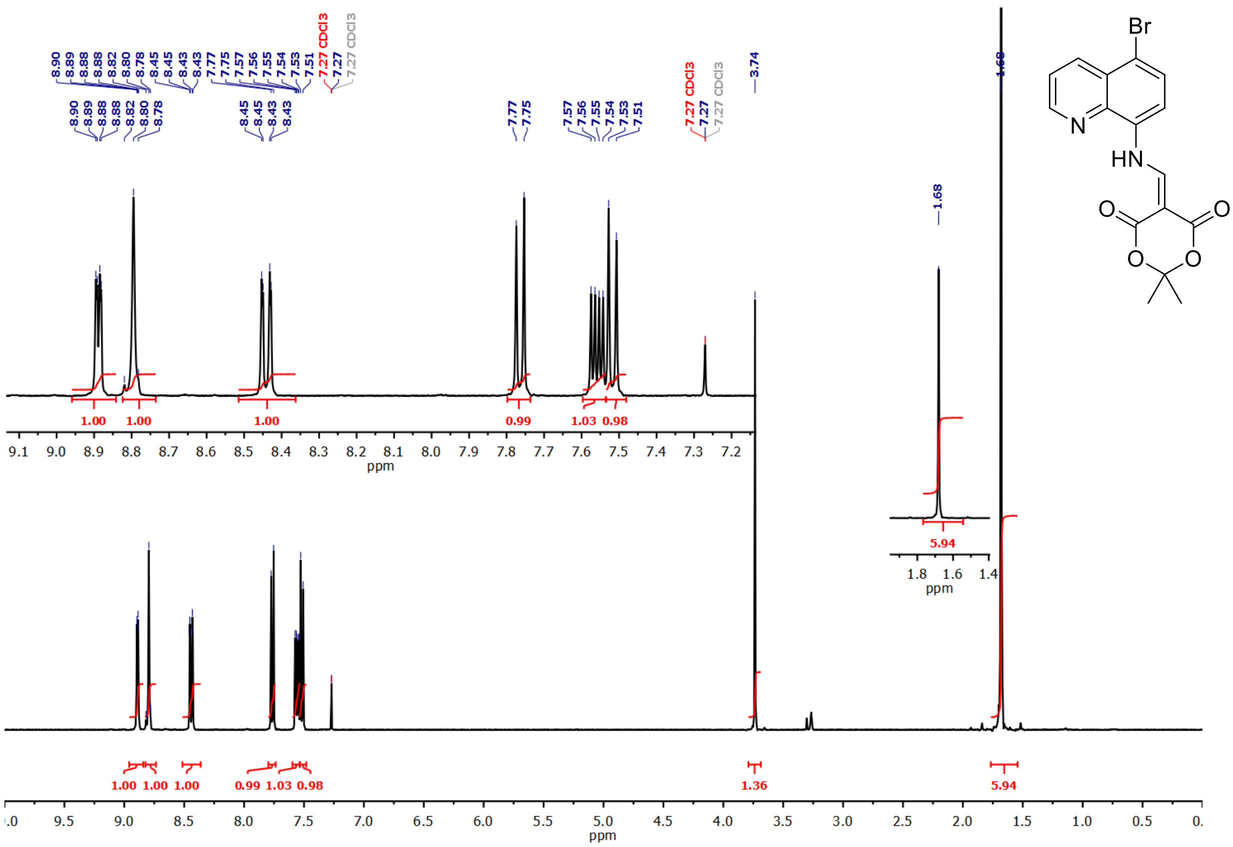

Fig. S21. ^{1}H NMR spectrum of the compound **16** ($CDCl_3$–CD_3OD (5:1 v/v), 400 MHz, 298 K).

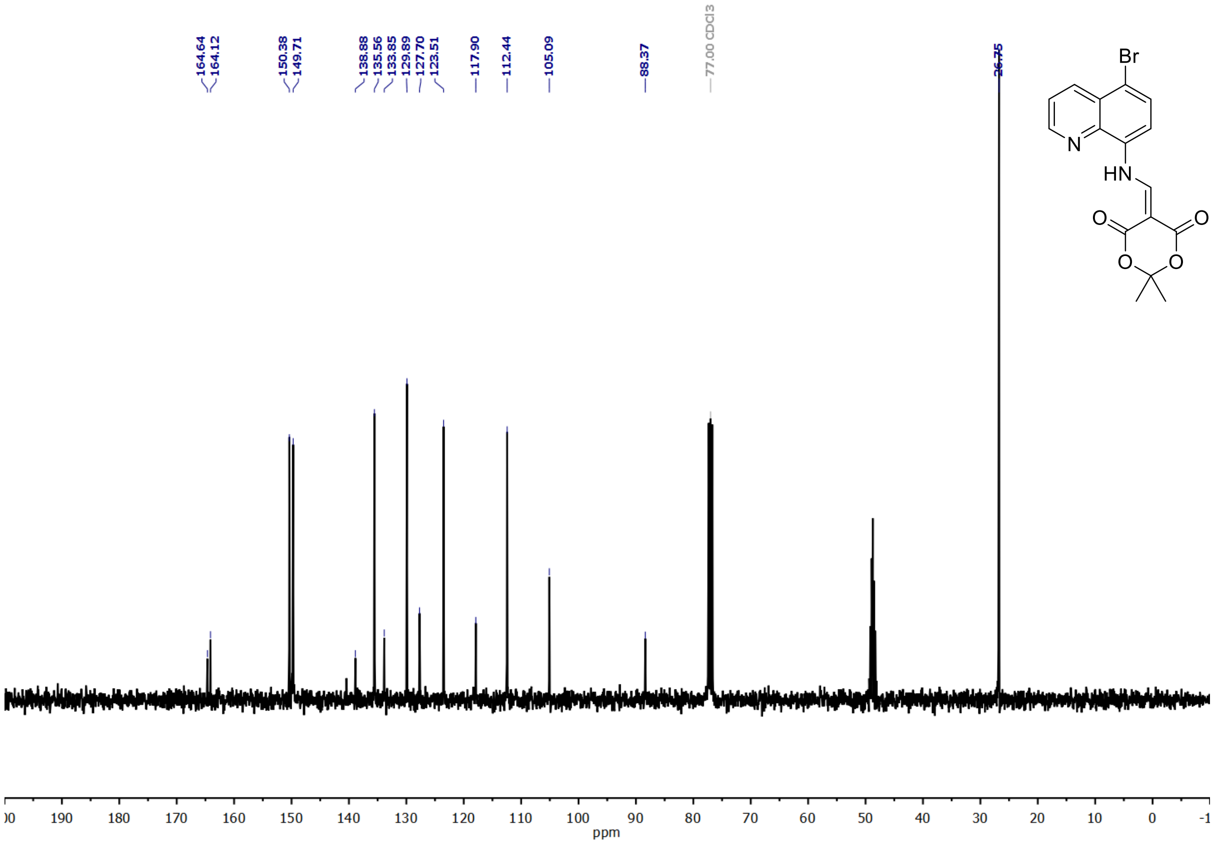

Fig. S22. ^{13}C NMR spectrum of the compound **16** ($CDCl_3$–CD_3OD (5:1 v/v), 100.6 MHz, 298 K).

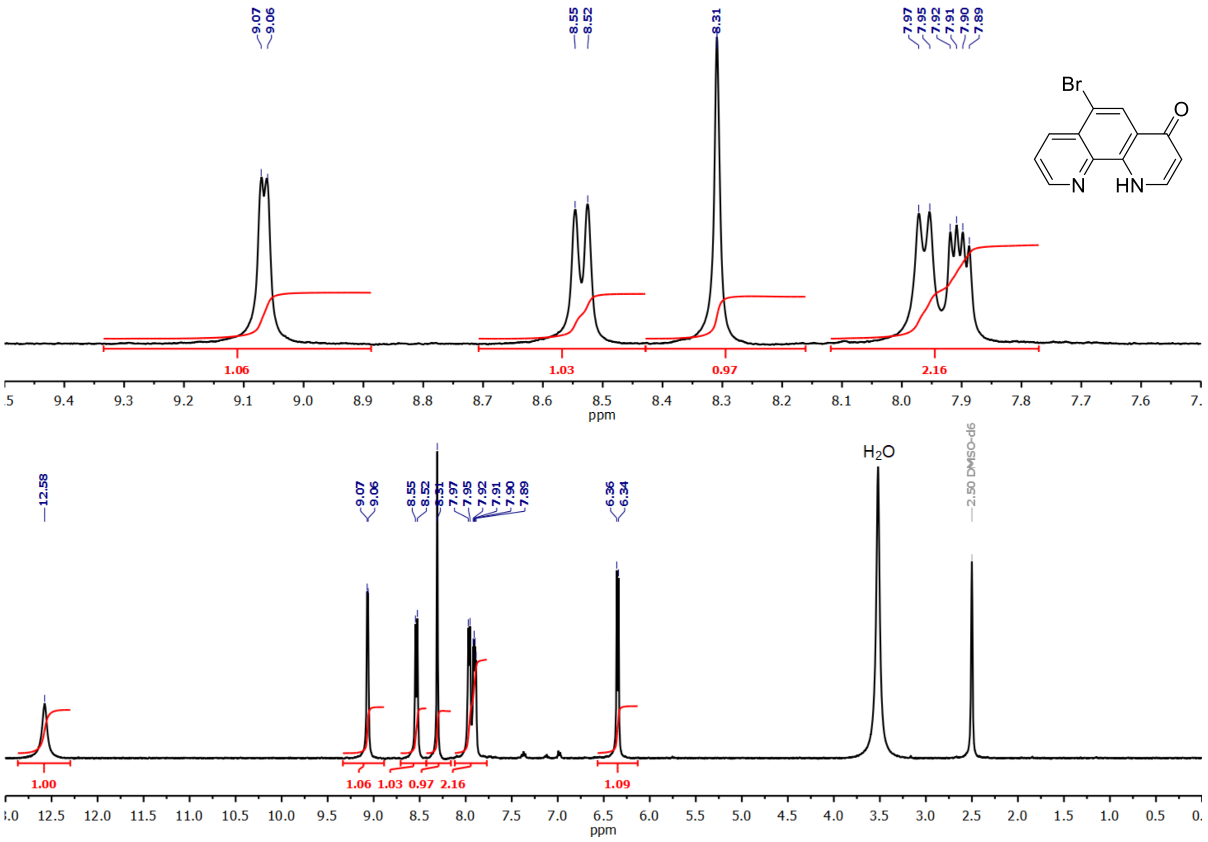

Fig. S23. ^{1}H NMR spectrum of the compound **17** (DMSO-D6, 400 MHz, 298 K).

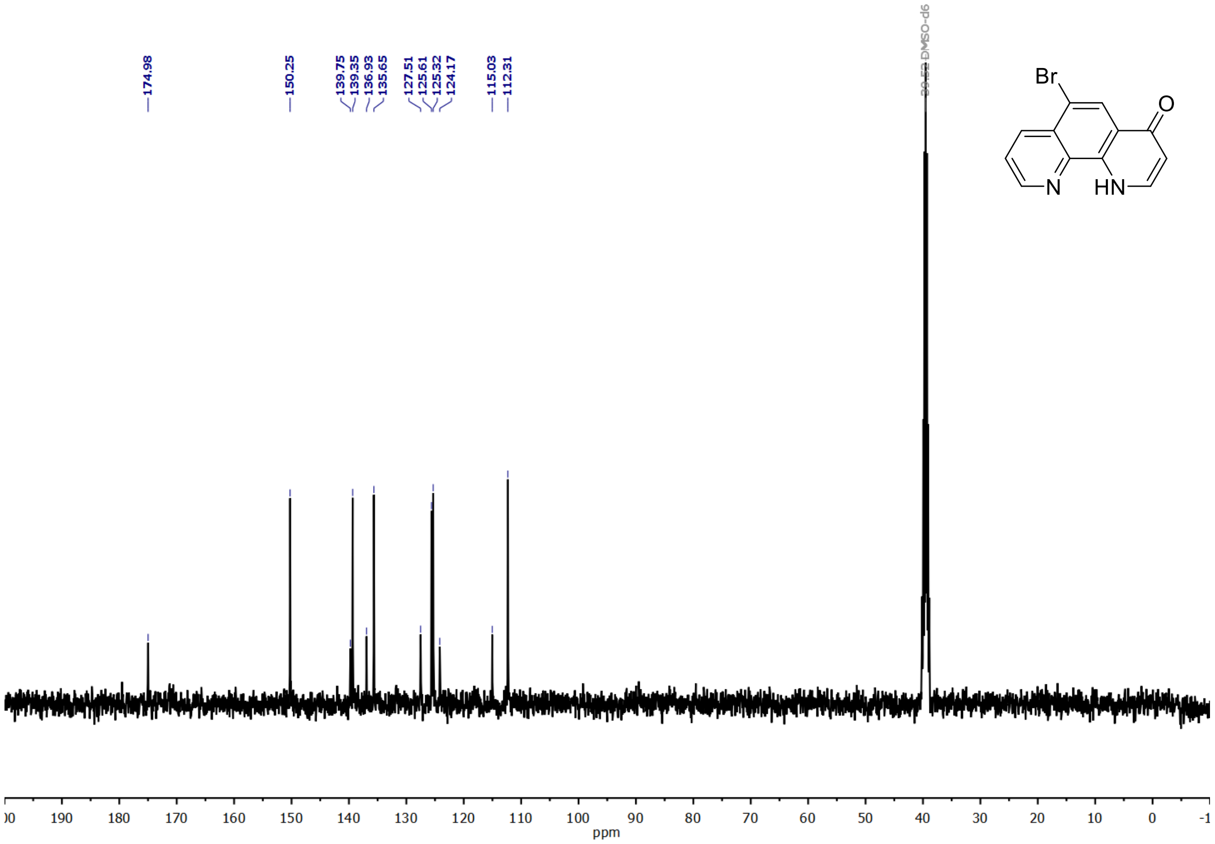

Fig. S24. ^{13}C NMR spectrum of the compound **17** (DMSO-D6, 100.6 MHz, 298 K).

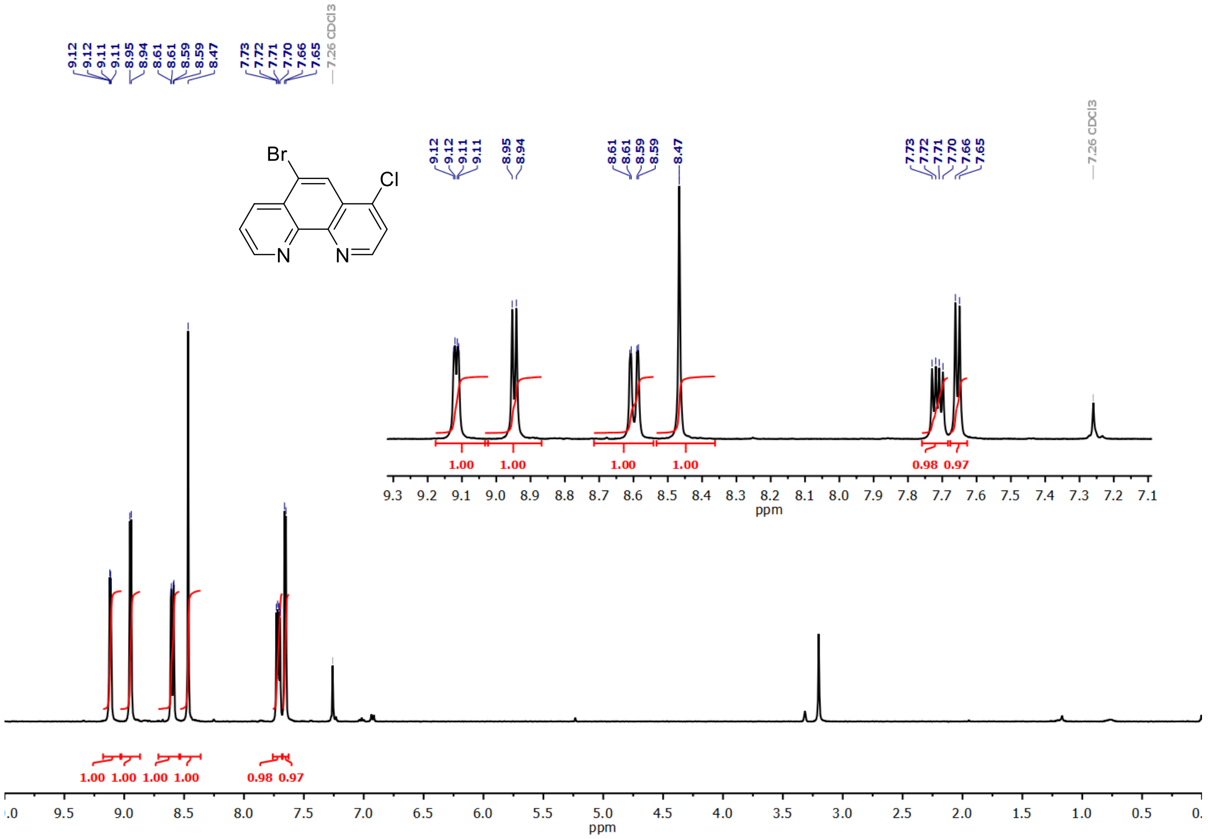

Fig. S25. ^{1}H NMR spectrum of the compound **18** ($CDCl_3$–CD_3OD (5:1 v/v), 400 MHz, 298 K).

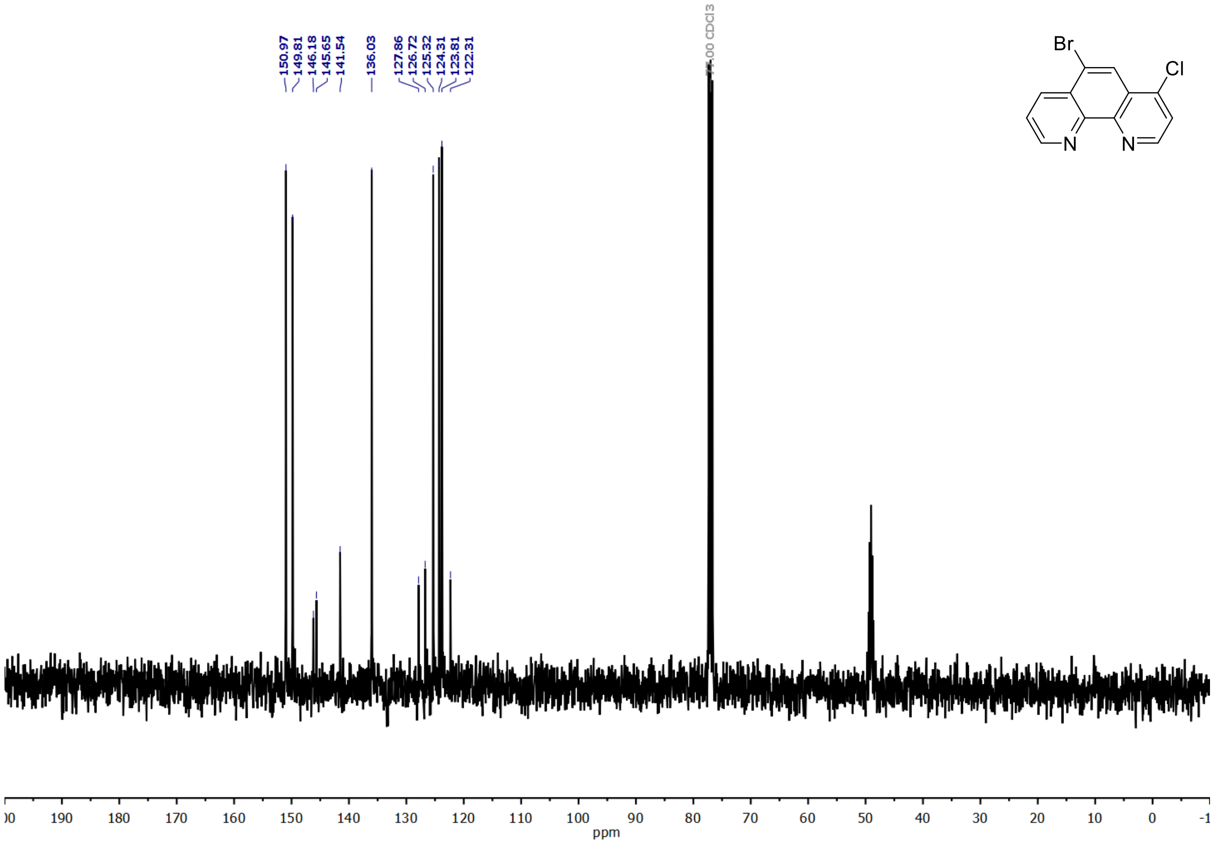

Fig. S26. ^{13}C NMR spectrum of the compound **18** ($CDCl_3$–CD_3OD (5:1 v/v), 100.6 MHz, 298 K).

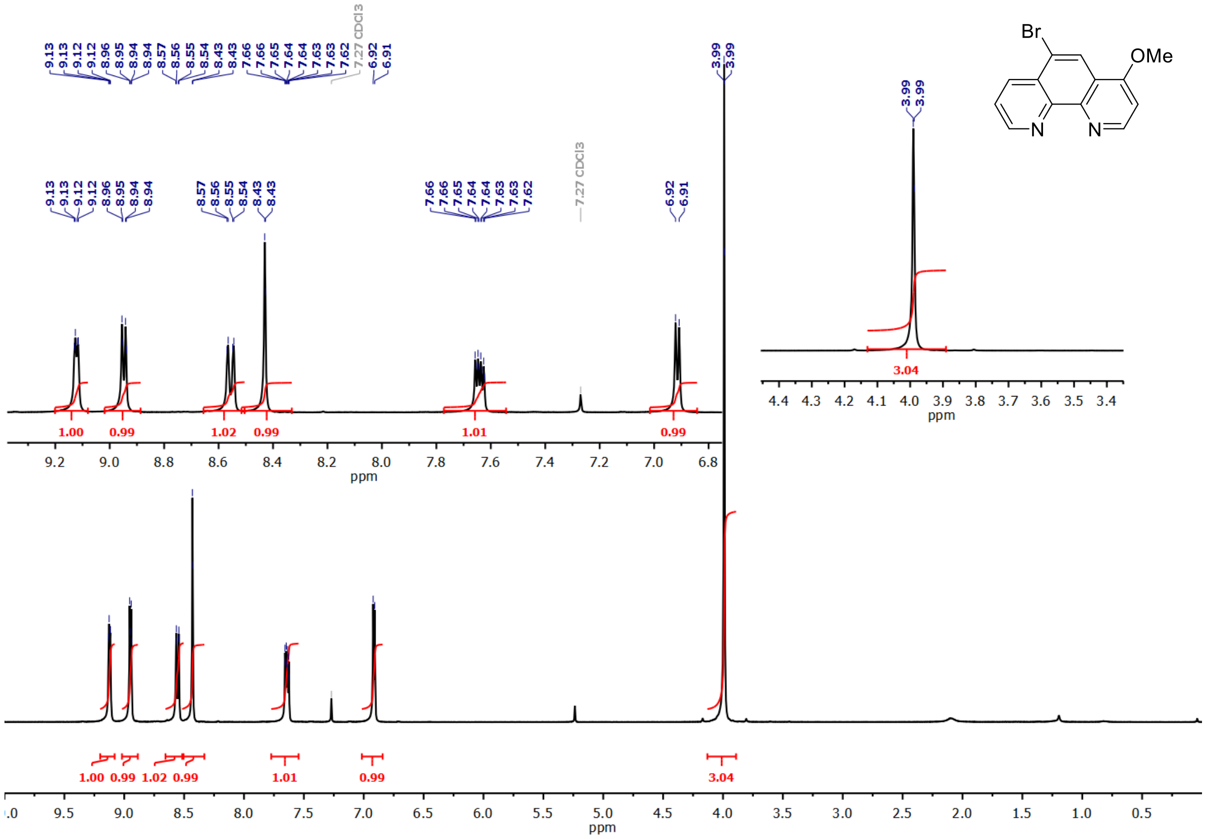

Fig. S27. ^{1}H NMR spectrum of the compound **19** ($CDCl_3$, 400 MHz, 298 K).

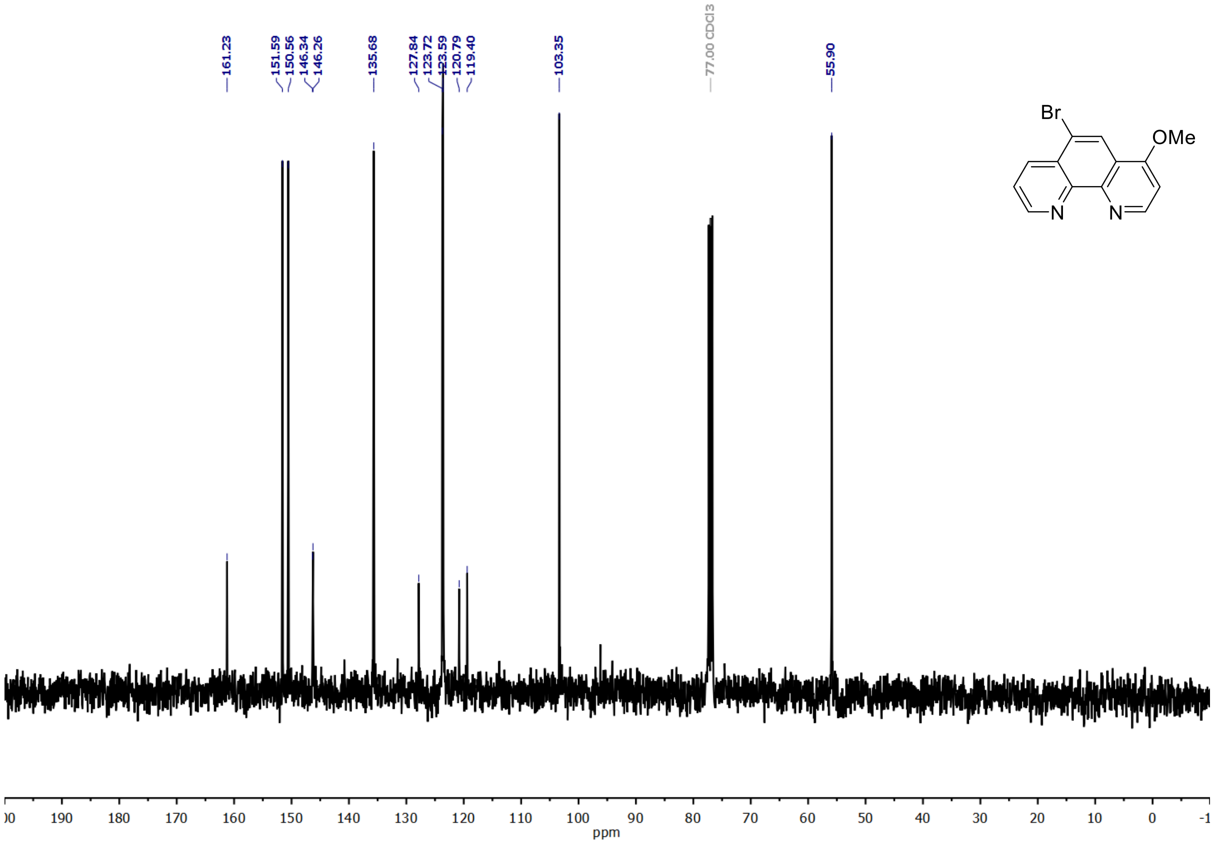

Fig. S28. ^{13}C NMR spectrum of the compound **19** ($CDCl_3$, 100.6 MHz, 298 K).

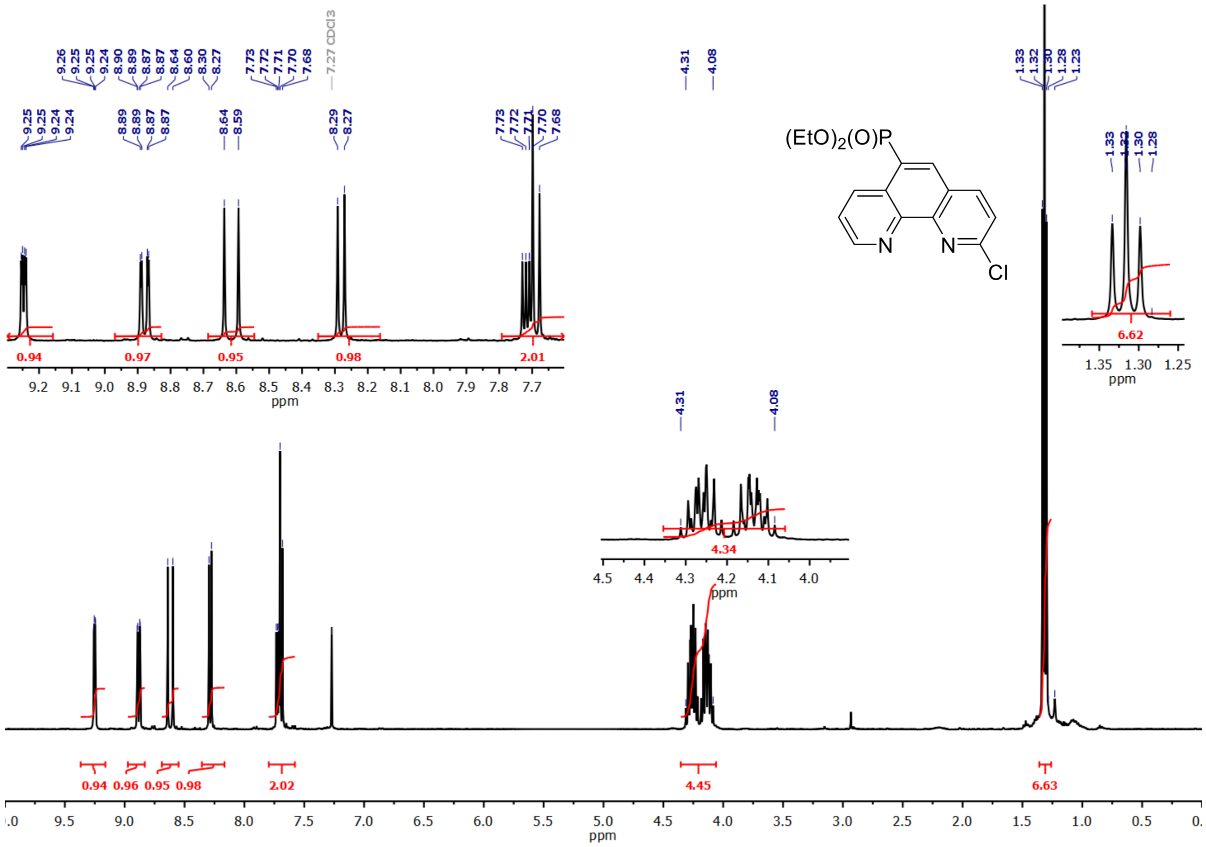

Fig. S29. ^{1}H NMR spectrum of the compound **20** ($CDCl_3$, 400 MHz, 298 K).

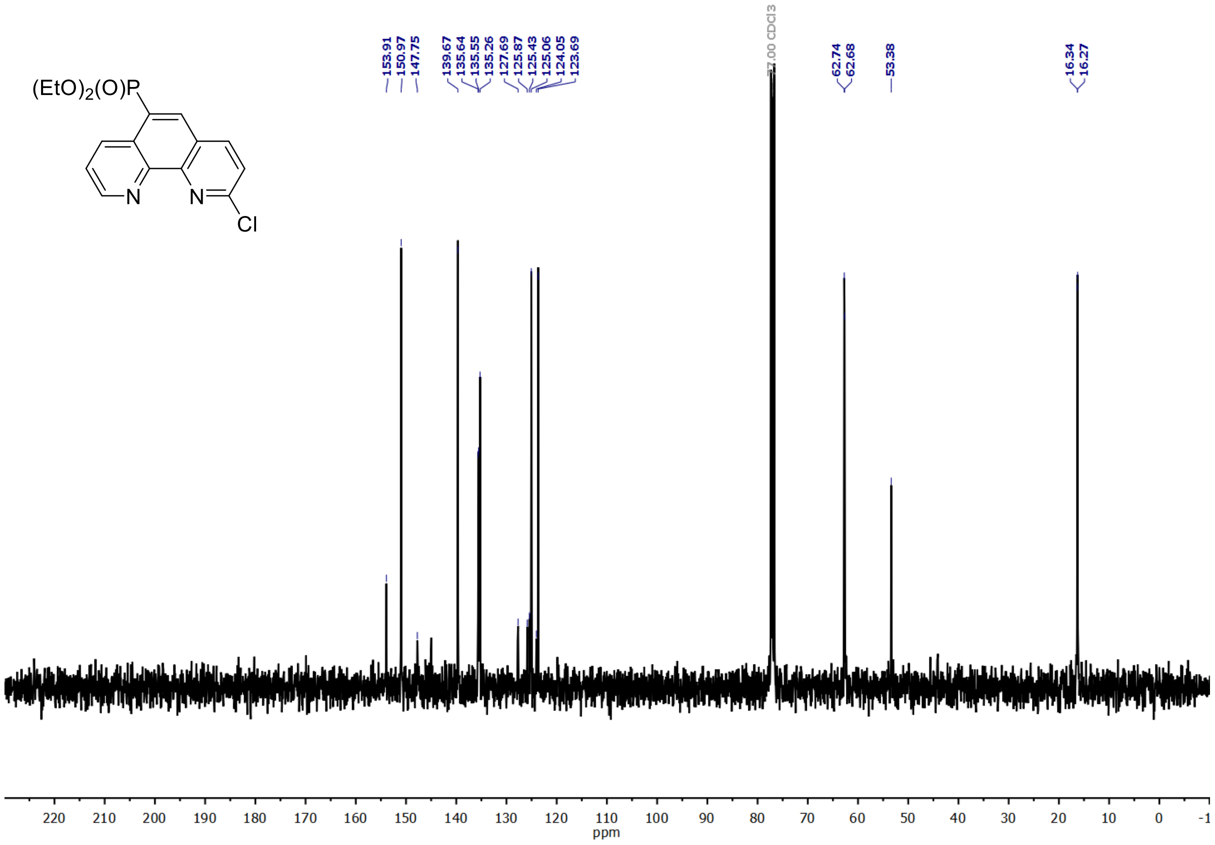

Fig. S30. ^{13}C NMR spectrum of the compound **20** ($CDCl_3$, 100.6 MHz, 298 K).

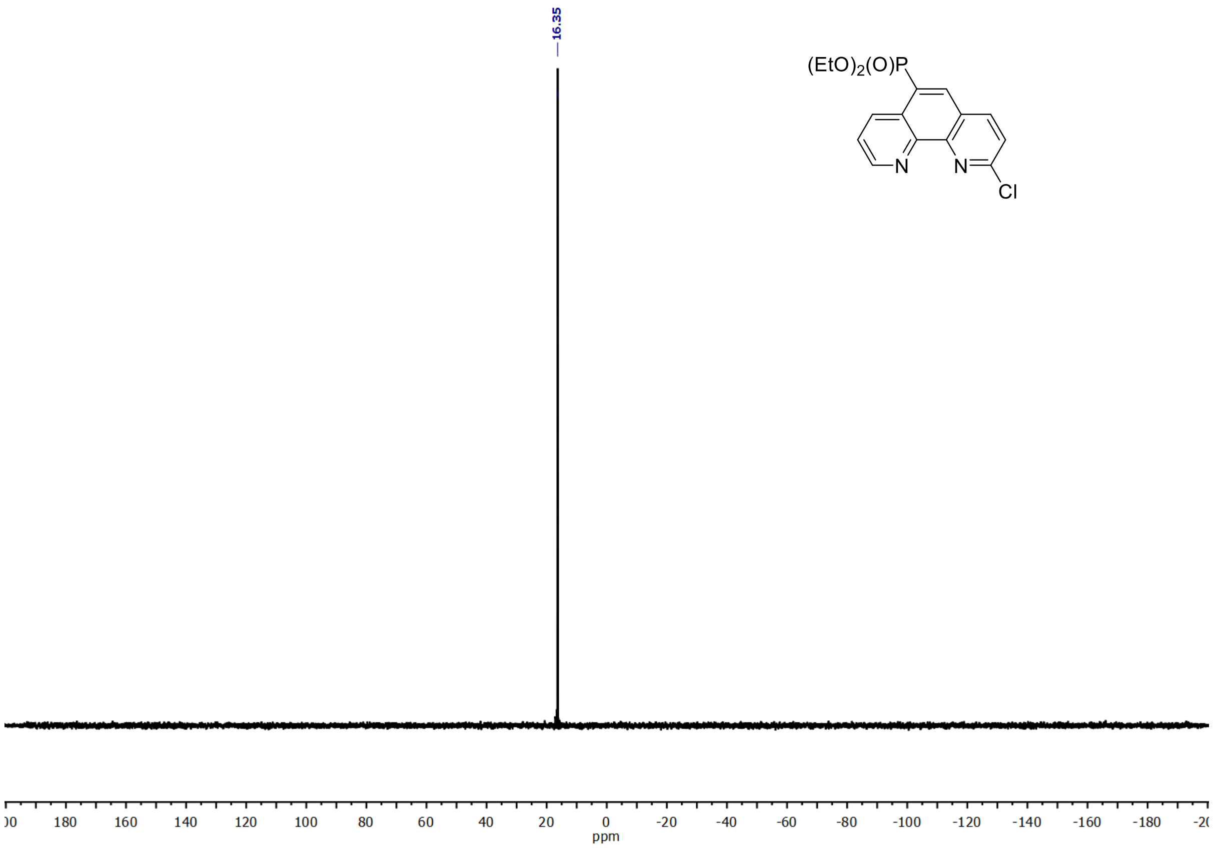

Fig. S31. $^{31}P\{^{1}H\}$ NMR spectrum of the compound **20** ($CDCl_3$, 162.5 MHz, 298 K).

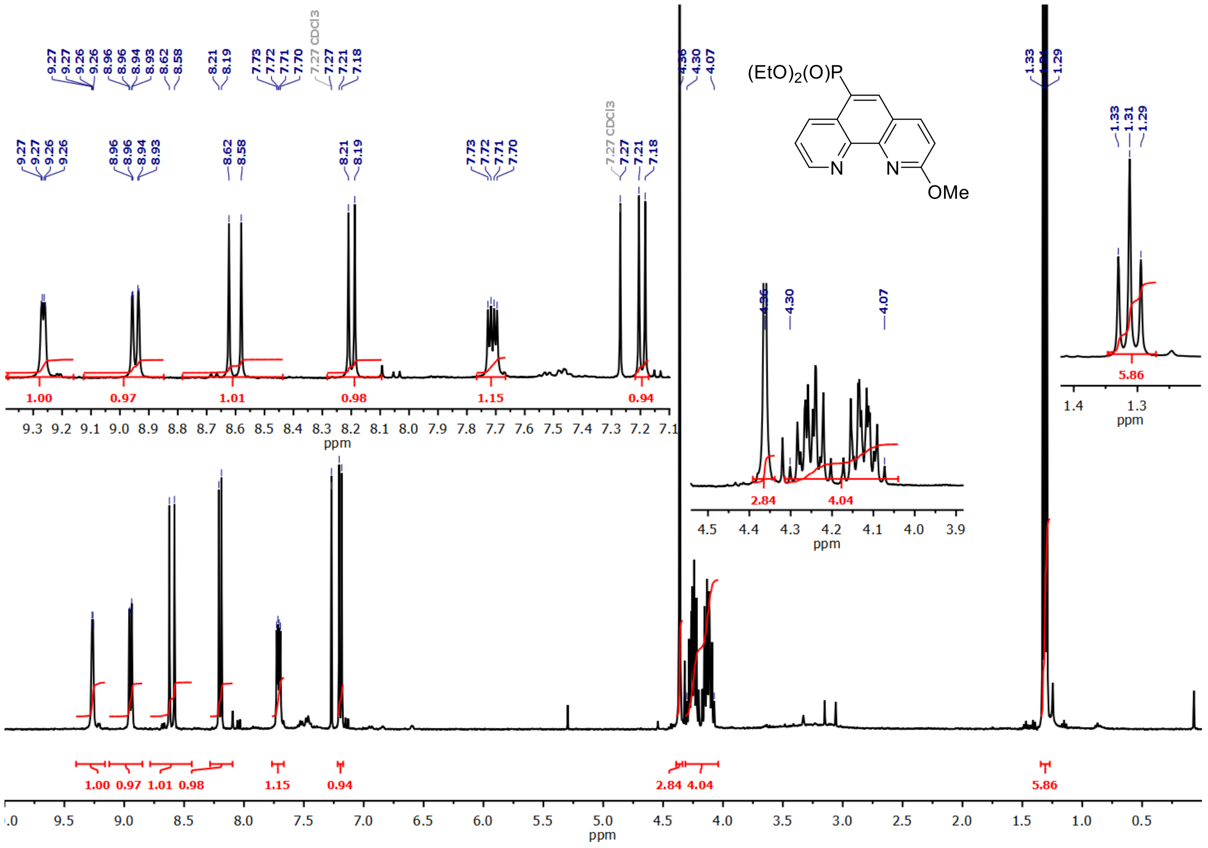

Fig. S32. ^{1}H NMR spectrum of the compound **22** ($CDCl_3$, 400 MHz, 298 K).

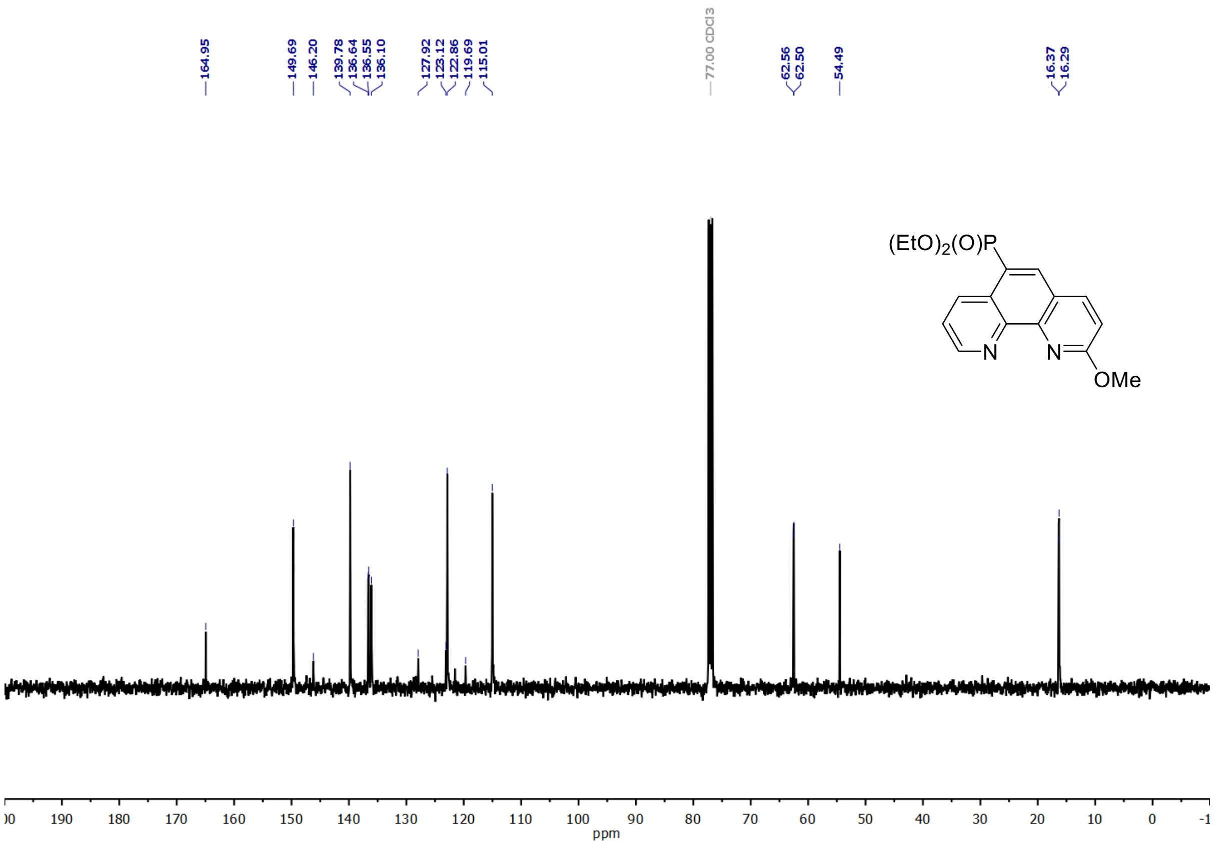

Fig. S33. ^{13}C NMR spectrum of the compound **22** ($CDCl_3$, 100.6 MHz, 298 K).

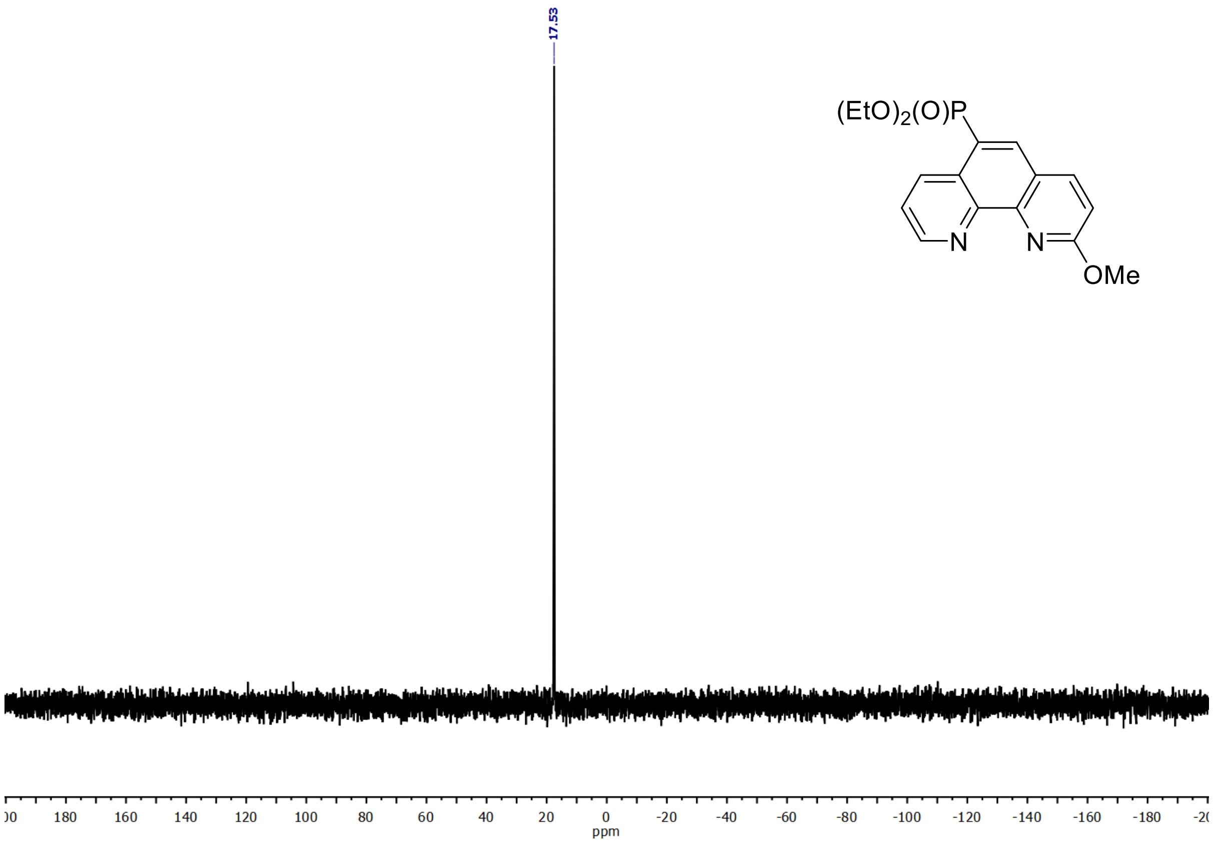

Fig. S34. $^{31}P\{^1H\}$ NMR spectrum of the compound **22** ($CDCl_3$, 162.5 MHz, 298 K).

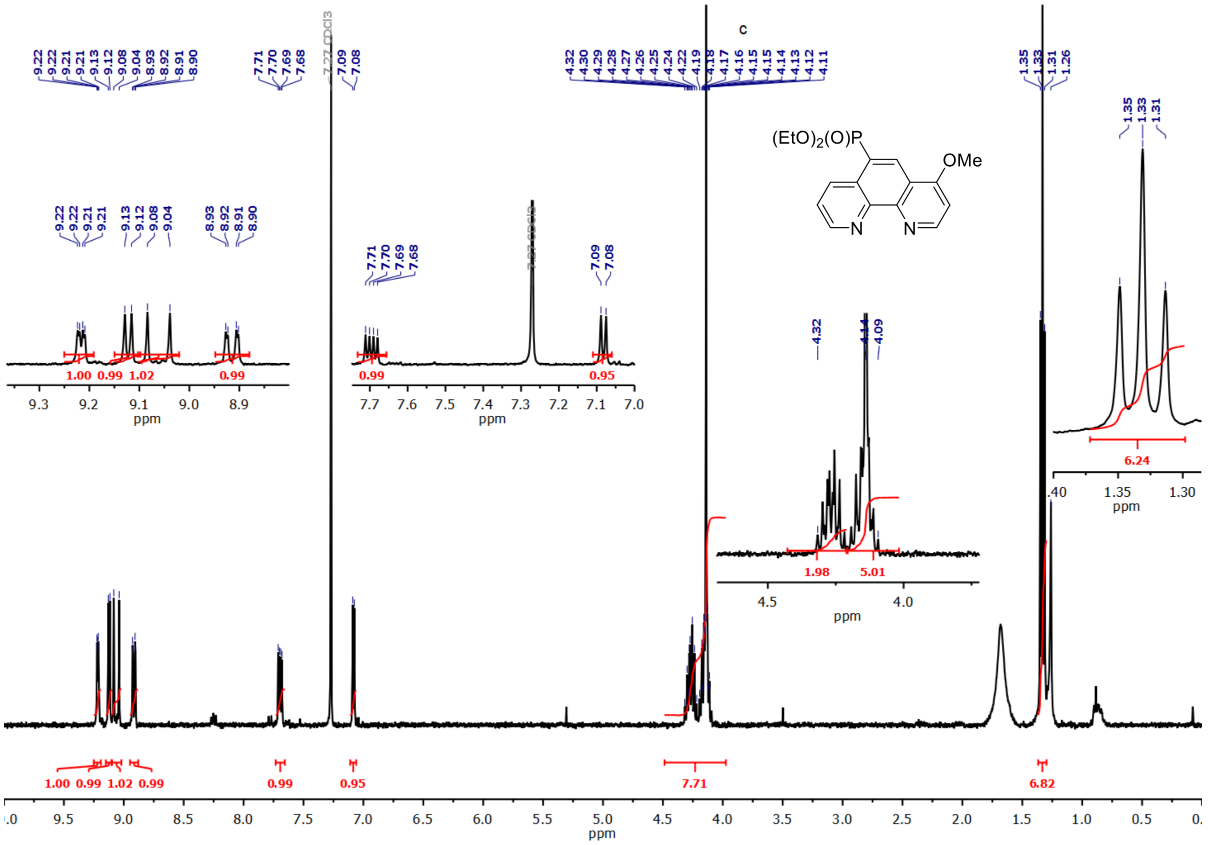

Fig. S35. ^{1}H NMR spectrum of the compound **23** ($CDCl_3$, 400 MHz, 298 K).

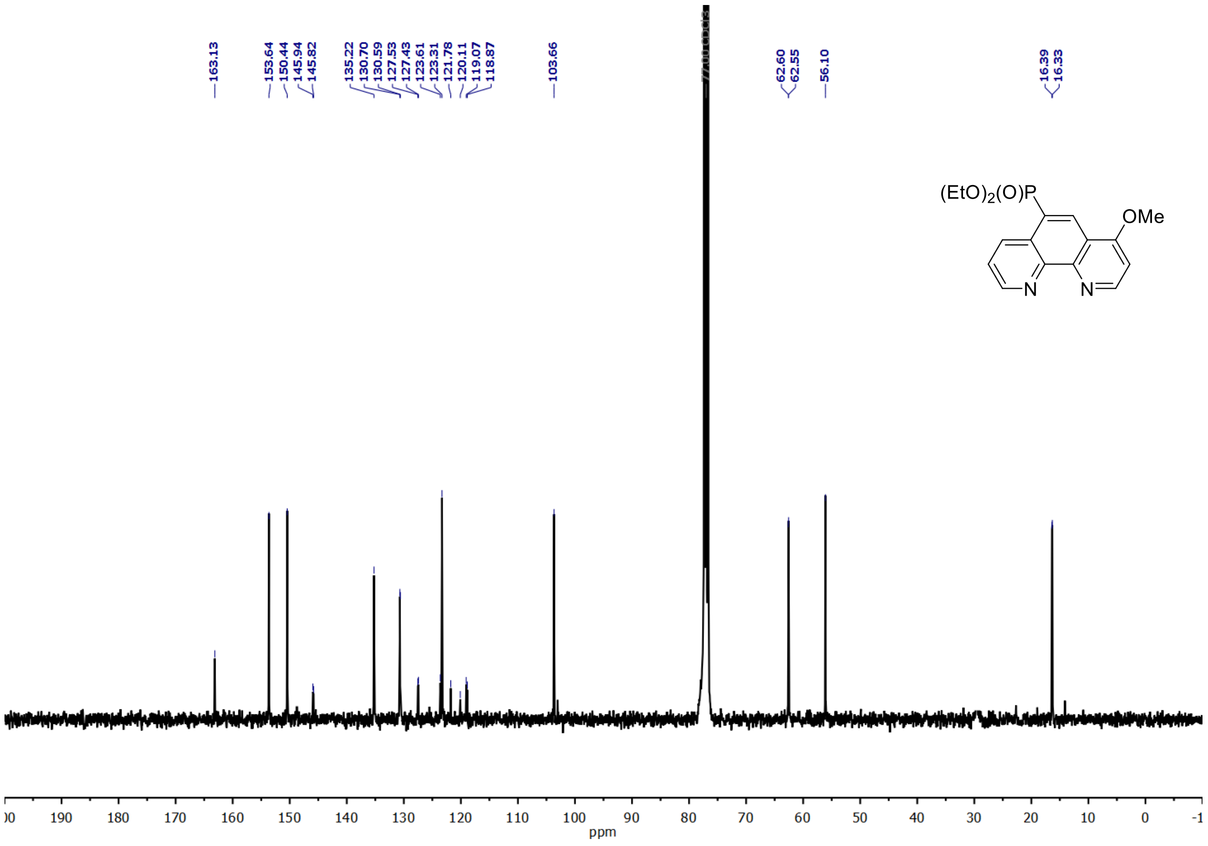

Fig. S36. ^{13}C NMR spectrum of the compound **23** ($CDCl_3$, 100.6 MHz, 298 K).

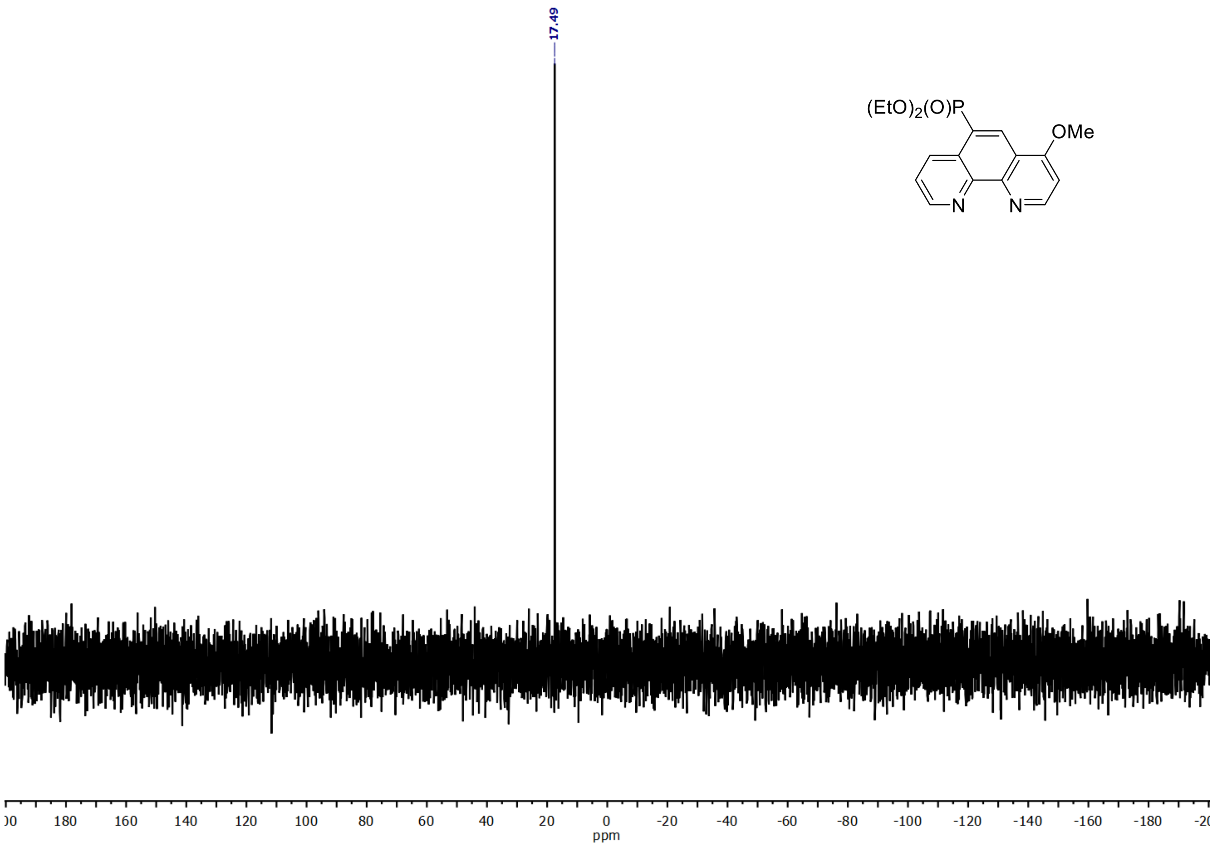

Fig. S37. $^{31}P\{^1H\}$ NMR spectrum of the compound **23** ($CDCl_3$, 162.5 MHz, 298 K).
